# Genome-wide identification of m^6^A methyltransferase genes and m^6^A modification participates in the response to cold stress in rice

**DOI:** 10.3389/fpls.2026.1804596

**Published:** 2026-04-13

**Authors:** Shihuan He, Jinxi Xiang, Rui Wang, Zichan Ye, Yue Zhang, Yunxia Fang, Xiaoqin Zhang, Jian Zhang, Xiaoguang Chen, Dawei Xue

**Affiliations:** 1 College of Life and Environmental Sciences, Hangzhou Normal University, Hangzhou, China; 2 State Key Laboratory of Rice Biology and Breeding, China National Rice Research Institute, Hangzhou, China

**Keywords:** cold stress, m^6^A, m^6^A me-RIP sequence, rice, RNA modification

## Abstract

RNA modification is crucial for the post-transcriptional regulatory mechanism that plays a pivotal role in determining RNA structure and function. Among these, N6-methyladenosine (m^6^A) represents the most abundant one in eukaryotic mRNA. In plants, m^6^A modification is catalyzed by a complex comprising multiple methyltransferase components. In this study, bioinformatic analyses were employed to characterize the genes of m^6^A methyltransferases (m^6^A writers), including their physicochemical properties, structures, *cis*-acting elements, chromosomal distributions, phylogenetic relationships, and predicted protein structures. Moreover, qRT-PCR and LC-MS/MS were utilized to investigate the expression patterns of m^6^A writer genes as well as the m^6^A abundance in total RNA from rice seedlings under low-temperature conditions. Additionally, m^6^A me-RIP sequencing was performed to explore changes in the m^6^A profile of mRNA in rice under cold stress. Collectively, our findings revealed the involvement in the regulation of mRNA m^6^A modification under cold stress in rice.

## 1 Introduction

RNA modification is one of the post transcriptional regulatory mechanisms, which have been found in eukaryotes, archaea, and bacteria widespreadly. Over 170 types of RNA modification have been identified which are widely distributed on various types of RNA such as messenger RNA (mRNA), transfer RNA (tRNA), ribosomal RNA (rRNA), small non coding RNA, and long non-coding RNA, affecting the stability, structure, function, processing, and regulation of RNA molecules, playing important roles in RNA metabolism such as splicing, polyadenylation, transport, localization, translatability, and stability (Andrea et al., 2024; Zhou et al., 2020). It has been known that N6-methyladenosine (m^6^A) is the most abundant internal modification in eukaryotic mRNAs among all known modifications (Jia et al., 2013). The m^6^A mark is installed, removed, and interpreted by methyltransferases (known as “writers”), demethylases (referred to as “erasers”) (Shi et al., 2019), and RNA‐binding proteins (referred to as “readers”) (Han et al., 2021), respectively. These proteins work together to regulate m^6^A homeostasis.

In model plant Arabidopsis, m^6^A modifications in mRNA are installed by a methyltransferase complex comprising adenosine methyltransferase A (MTA) (Bodi et al., 2012), which is the ortholog of human methyltransferase-like 3 (METTL3); adenosine methyltransferase B (MTB) (Yue et al., 2019), the ortholog of human METTL14; fkbp12 interacting protein 37 kDa (FIP37) (Shen et al., 2016); virilizer (VIR) (Hu et al., 2021); and the E3 ubiquitin ligase HAKAI (Růžička et al., 2017). Those components play crucial roles in mRNA m^6^A homeostasis, resulting in the regulation of Arabidopsis growth and development. For instance, null alleles of *MTA*, *MTB*, *FIP37*, and *VIR* arrest at the globular stage of embryonic development, while *HAKAI* mutants were viable but exhibited a dwarfing phenotype. Moreover, FIONA1 has been known as a small nuclear RNA (snRNA) m^6^A methyltransferase, involving in the regulation of m^6^A level in U6 snRNA and a small amount of poly(A)^+^ RNA (Wang C. et al., 2022). The homolog of human METTL4, adenosine methyltransferase C (MTC), was characterized as a U2 snRNA methyltransferase for N6-2’-dimethyladenosine, participating in the control of flowering time (Luo et al., 2022). In rice, eukaryotic translation initiation factor 3 subunit H (OseIF3h) has been identified as the initiation factor of the *OsMTA* gene. Defective *OseIF3h* mutant led to reduction of seed-setting rate as well as growth retardation, suggesting that RNA m^6^A modification mediated via OsMTA plays a vital role in safeguarding rice yields (Huang et al., 2021). Furthermore, rice enhanced downy mildew 2 like (OsEDM2L), which is an N6 adenine methyltransferase like domain containing protein, involved in the m^6^A generation of *eternal tapetum 1* (*EAT1*) transcript, regulating tapetal programmed cell death (PCD) and male sterility. Knock-out mutant of *OsEDM2L* exhibits delayed tapetal PCD as well as impaired pollen development (Ma et al., 2021). Besides, m^6^A modification plays a crucial role in response to abiotic stress. All the Arabidopsis mutants of m^6^A writer genes are salt sensitive (Hu et al., 2021; Cai et al., 2024). In rice, it has been reported that m^6^A modification is involved in the response to salt stress, leading to the differential m^6^A levels in salt stress response genes like *HAK4*, *CIPK06*, *RBOHH*, *Myb10*, and *ERF067* (Chen et al., 2022). Similar results were also investigated in sweet sorghum, m^6^A modification is up-regulated on the transcripts of salt-tolerance-related genes like *SbIAR4* and *SbNRT1.5*, accompanied by increased transcription levels of those genes (Zheng et al., 2021).

Rice serves as the staple food for over half of the world’s population. As one of the most important crops, its yield is severely constrained by cold stress, which constitutes the primary environmental stressor that hinders the growth and development of rice plants, ultimately leading to a decline in grain yield (Ma et al., 2023). Specifically, continuous intense cold stress triggers excessive intracellular reactive oxygen species (ROS), which cause a series of cytotoxic effects, including cell death, lipid peroxidation, photosynthetic rate reduction, growth retardation, and even seedling death or spikelet sterility (Xu et al., 2020). To counteract the damage caused by low temperatures, plants activate a series of molecular mechanisms after exposure to a non-lethal temperature to enhance their tolerance, a process known as cold acclimation. Some metabolites (such as sugars and proline) begin to accumulate as osmolytes and cryoprotectants to prevent damage to the organism caused by chilling injury (Xin and Browse, 1998; Rahemi et al., 2016). Besides, multiple hormones are involved in cold response, including phytohormones such as auxin (Rahman, 2013), gibberellin (Achard et al., 2008), abscisic acid (Guan et al., 2023), cytokinin (Jeon et al., 2010; Maruyama et al., 2014), ethylene (Shi et al., 2012; Zhang and Huang, 2010), and Methyl Jasmonate (Seoa et al., 2020). Moreover, cold tolerance genes like *qLT3-1* (Fujino et al., 2008), *COLD1* (Ma et al., 2015), *qCTS-9* (Zhang et al., 2014), *GSTZ2* (Kim et al., 2011), *HAN1* (Mao et al., 2019), *Ctb1* (Saito et al., 2010), and *CTB4a* (Zhang et al., 2017) have been characterized in rice, playing crucial roles in response to cold stress. Interestingly, RNA modification, particularly pseudouridine (Ψ), has been reported to participate in the response to cold stress in rice. Pseudouridine synthase (OsPUS1) loss-of-function mutant leads to the reduction of Ψ modification in RNA as well as abnormal chloroplast development and albino seedling phenotype under low-temperature conditions (Wang Z. et al., 2022).

In order to better understand the m^6^A writer genes and the role of mRNA m^6^A modification response to cold stress in rice, bioinformatic analyses, including the physicochemical properties, gene and protein structures, *cis*-acting elements, expression profiles, chromosomal distribution, and phylogeny of m^6^A methyltransferase genes, as well as the expression pattern of m^6^A writer genes and m^6^A me-RIP sequencing, were performed. The results will help to enrich the understanding of the m^6^A writer genes and provide a theoretical basis for further studying the function of m^6^A in the cold stress response of rice.

## 2 Materials and methods

### 2.1 Phylogenetic and characteristic analyses of rice m^6^A writers

The protein sequences of the m^6^A writers in Arabidopsis (*Arabidopsis thaliana*) were retrieved from the TAIR database (https://www.arabidopsis.org) and served as the benchmark sequences. Following this, the protein sequences of m^6^A writers in *Oryza sativa*, *Sorghum bicolor*, *Zea mays*, *Arabidopsis thaliana*, *Phaseolus vulgaris*, and *Solanum lycopersicum* were identified through the BlastP tool from the Phytozome database (https://phytozome-next.jgi.doe.gov) refer to Liu et al (Liu et al., 2024). After inputting the species name (*Arabidopsis thaliana TAIR10*) and the characterized m^6^A writer protein sequence into the online tool Phytozome for BLAST, the genes with numerical values in the “ortho” column of the Protein Homologs table were selected as the orthologs corresponding to rice. The protein sequences were then aligned employing the ClustalW method in MEGA11. To investigate their evolutionary relationships, a phylogenetic tree was constructed with the Neighbor-Joining (NJ) method and 1000 bootstrap replicates, based on the identified MT-A70 m^6^A writer protein sequences from the aforementioned species. Subsequently, the phylogenetic tree was visualized with ChiPlot (https://www.chiplot.online/).

### 2.2 Analyses of m^6^A writer gene structure, conserved motifs, collinearity relationship, and *cis*-elements

Conserved motifs of the proteins were analyzed via MEME (https://meme-suite.org/meme/tools/meme). The settings were configured with ‘any number of repetitions’ (anr) for site distribution, the number of motifs was specified as 10, and all other optional parameters were kept at their default settings. The outcomes of the conserved domain analysis were then depicted using Chiplot. The collinear relationships among the m^6^A writer genes in rice were predicted by the One Step MCScanX-Super Fast program in TBtools. For the prediction of *cis*-elements, the sequences of the 2000 bp upstream from the start codons of the m^6^A writer genes were extracted and submitted to the PlantCARE database (https://bioinformatics.psb.ugent.be/webtools/plantcare/html/).

### 2.3 Protein physicochemical property, subcellular localization, secondary and tertiary structure prediction

Protein physicochemical properties, including GRAVY (Grand Average of Hydropathicity), molecular weight, and theoretical pI (isoelectric point), were analyzed by using ExPASy (https://web.expasy.org/protparam/). Subcellular localization was predicted by WoLF (https://wolfpsort.hgc.jp/). Conserved domains were analyzed using the CD-Search tool available at NCBI (https://www.ncbi.nlm.nih.gov/Structure/cdd/wrpsb.cgi) and the results were visualized with TBtools. Secondary as well as tertiary structure of m^6^A writer proteins were predicted via SOPMA (https://npsa-prabi.ibcp.fr/cgi-bin/npsa_automat.pl?page=npsa_sopma.html) and SWISS-model (https://swissmodel.expasy.org/), respectively.

### 2.4 Plant cultivation and cold stress treatment

For rice (ZH11) cultivation, the seeds were firstly sterilized by chlorine for 30 min. After thoroughly rinsing off the chlorine, the seeds were soaked in H_2_O and incubated at 30 °C for 3 days in the dark to promote germination. Subsequently, the seeds were transferred to 96-well plate in the climate chamber and cultured in Yoshida nutrient medium (YM) under long-day conditions (14 h light of 100 μmol m^-2^ s^-1^ intensity at 28°C, 10 h dark at 26°C, 70% relative humidity). YM is renewed every three days. For the cold treatment, 2-week-old seedlings were grown at 4°C for 6 h, then harvested in a 2 mL tube and rapidly frozen in liquid nitrogen.

### 2.5 RNA extraction and qRT-PCR

Total RNA was isolated from roots, stems, leaves, and 2-week-old seedlings by using Eastep Super Total RNA Extraction Reagent (Promega). The quantity and concentration of the extracted RNA were then evaluated by NanoDrop™ spectrophotometer (Thermo). For the synthesis of first-strand cDNA, a total of 1000 ng of RNA was utilized with an Oligo (dt) primer by HiScript III RT SuperMix (Vazyme). Quantitative PCR was conducted using Hieff qPCR SYBR Green Master Mix (Yeasen). Statistical analysis was performed by GraphPad Prism 8.0.2 software. Each sample had three biological replicates, while each biological replicate contained three technical replicates.

The transcript abundance of m^6^A writer genes including *OsMTA*, *OsMTB1*, *OsMTB2*, *OsMTB3*, *OsMTC*, *OsVIR*, *OsFIP37*, *OsHAKAI*, and *OsFIONA1* was analyzed by employing the primer pairs P226/P227, P230/P231, P234/P235, P240/P241, P242/P243, P252/P253, P246/P247, P254/P255, and P340/P341, respectively. *OsUBQ5* (Ubiquitin 5) was amplified using primers P78 and P79 as an internal reference gene. The results were calculated based on the 2^−ΔΔCT^ method. All primer sequences are detailed in **Supplementary Table 1**.

### 2.6 The detection of m^6^A abundance in total RNA

A total of 800 ng RNA underwent full digestion into single nucleotides within a 50 μL reaction buffer, which was composed of 10 mM Tris–HCl (pH 7.9), 1mM MgCl_2_, 0.1 mg mL^-1^ BSA, 0.4 units of benzonase (Sigma-Aldrich), 0.004 units of phosphodiesterase I (Sigma-Aldrich), and 0.04 units of shrimp alkaline phosphatase (NEB). Following a 10-hour incubation at 37 °C, the enzymatic reaction was halted, and the sample was filtered using an ultrafiltration tube (3 kDa cutoff; Pall). Subsequently, 2 μL aliquots of the sample were subjected to analysis by an ACQUITY Premier liquid chromatography system coupled with the Xevo Absolute mass spectrometer, and data processing was performed with MassLynx V4.2 software. The following mass transitions were monitored: m/z 268.1 to 136 (A, adenosine); m/z 282.12 to 150 (m^6^A, N^6^-methyladenosine) (Liu et al., 2024). Standard solutions of A: 0.8, 4, 8, 40, 200, 400, 2000, and 10000 ng/ml; m^6^A: 0.08, 0.4, 0.8, 4, 20, 40, 200, and 1000 ng/ml were used for quantification. The ratios of m^6^A to A were calculated based on the calibrated concentrations.

### 2.7 m^6^A mRNA immunoprecipitation sequencing

mRNA m^6^A sequencing was performed via the RNA immunoprecipitation method by Seqhealth Technology Co., Ltd (Wuhan, China). Briefly, polyadenylated RNA was enriched from the total RNA using VAHTS mRNA Capture Beads (Vazyme), following the manufacturer’s protocol. PolyA+ RNA was fragmented into approximately 100 nt fragments by treatment with 20 mM ZnCl_2_ at 95°C for 5 min. A portion (10%) of the RNA fragment was reserved as “Input”, while the remainder was incubated with m^6^A antibody (Synaptic Systems). Subsequently, mRNA as well as the m^6^A IP cDNA libraries were prepared by KC™ Digital mRNA library Prep Kit (Seqhealth) and sequenced on the DNBSEQ-T7 platform (MGI).

## 3 Result

### 3.1 Genome-wide identification and evolutionary analyses of mRNA m^6^A writer genes in rice

**Figure 1:**
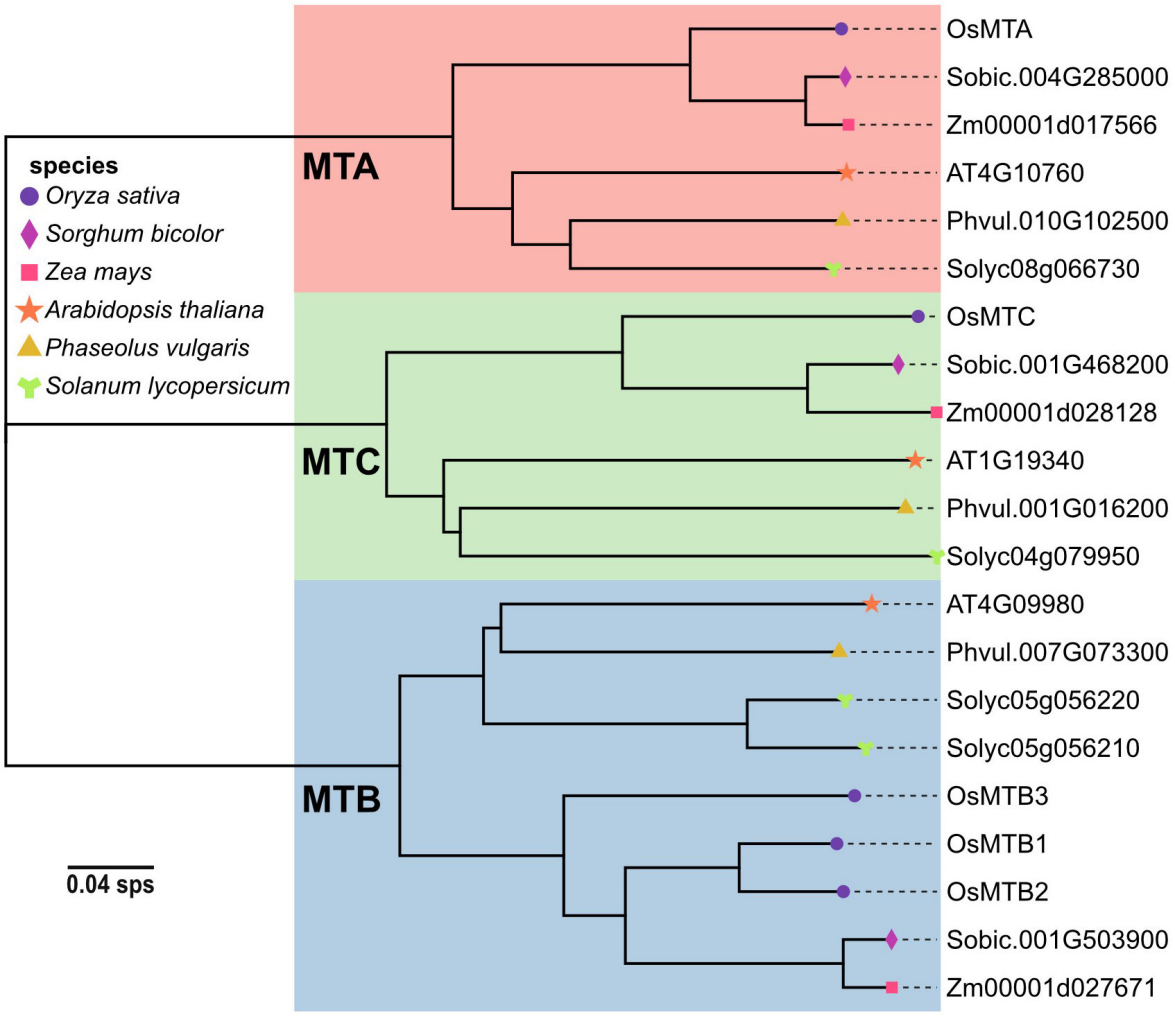
Phylogenetic analysis of m^6^A writers within MT-A70 family in *Oryza sativa*, *Sorghum bicolor*, *Zea mays*, *Arabidopsis thaliana*, *Phaseolus vulgaris*, and *Solanum lycopersicum*. The phylogenetic tree was constructed based on the amino acid sequences by using MEGA11 software with the Neighbor-Joining algorithm and 1,000 bootstrap replicates.

To discover m^6^A writer genes in particular plants, the sequences of established writer proteins, including MTA, MTB, MTC, FIP37, VIR, HAKAI, and FIONA1, were used from the model plant Arabidopsis as queries in BLASTp searches against the genomes of rice (*Oryza sativa*), sorghum (*Sorghum bicolor*), corn (*Zea mays*), common bean (*Phaseolus vulgaris*), and tomato (*Solanum lycopersicum*) in the Phytozome V13 database. The genome and the amino acid information could be found in **Supplementary Table 2**. A phylogenetic tree was constructed using the Neighbor-Joining method to reconstruct the evolutionary relationships among the writer candidates within the MT-A70 family from five economic plants and Arabidopsis (**Figure 1**). MT-A70 m^6^A writers were categorized into three clades (MTA, MTB, and MTC) according to their structures. Each plant species possessed at least one homolog within each clade, with the MTB family being the most abundant (totaling 9 candidates). Among the six plants, rice retained the largest number of m^6^A methyltransferase candidates. Interestingly, rice encoded three MTBs, implying that they may exhibit functional redundancy or divergence.

In rice, the lengths of the amino acids of the MT-A70 members ranged from 427 to 1013. The estimated molecular weights were 49.39 kDa to 113.61 kDa, and their theoretical isoelectric points (pIs) spanned from 6.17 to 8.49. Additionally, the VIR candidate had the longest sequence, which was 2128 amino acids with a theoretical molecular weight of 233.75 kDa, and a pI of 5.16. On the contrary, the candidates within WTAP, HAKAI, and FIONA1 were shorter, comprising 352, 502, and 466 amino acids, respectively. Their molecular weights were projected to be 39.36,53.12, and 50.74 kDa, with pIs ranging from 5.11 to 7.58. Notably, the isoelectric points of all candidates except OsMTC and OsFIONA1 were less than 7.0, indicating that most of the m^6^A writers were possibly negatively charged in rice cells. Moreover, the predicted Grand Average of Hydropathicity (GRAVY) values were negative, ranging from -1.188 to -0.101, suggesting that these proteins were likely to be hydrophilic. Predictions of subcellular localization for rice m^6^A modification enzymes indicated that OsMTA localized to the nucleus or cytoplasm; Other MTA-70 members, OsHAKAI, and OsFIONA1 localized to the nucleus; while OsFIP37 and OsVIR localized to the nucleoplasm and plastid, respectively (**Table 1**).

**Table 1:** Characteristics of predicted m^6^A writer candidate genes in *Oryza sativa*.

Family	Gene name	Gene ID (phytozome)	Amino acid length	Isoelectric point	Molecular weight (kDa)	GRAVY	Subcellular localization prediction	Orthologous gene ID in A. thaliana
MT- A70	*OsMTA*	LOC_Os02g45110	706	6.76	77.80	-0.456	nucleus/ cytoplasm	AT4G10760
	*OsMTB1*	LOC_Os01g16180	764	6.75	113.61	-0.940	nucleus	AT4G09980
	*OsMTB2*	LOC_Os03g05420	753	6.75	83.59	-0.934	nucleus	
	*OsMTB3*	LOC_Os10g31030	1013	6.17	113.61	-1.188	nucleus	
	*OsMTC*	LOC_Os03g10224	427	8.49	49.39	-0.550	nucleus	AT1G19340
WTAP	*OsFIP37*	LOC_Os06g27970	352	5.11	39.36	-0.773	nucleoplam	AT3G54170
VIR	*OsVIR*	LOC_Os03g35340	2128	5.16	233.75	-0.101	plastid	AT3G05680
FIONA1	*OsFIONA1*	LOC_Os02g02880	466	7.58	50.74	-0.151	nucleus	AT2G21070
HAKAI	*OsHAKAI*	LOC_Os10g35190	502	6.70	53.12	-0.522	nucleus	AT5G01160

### 3.2 Chromosomal distribution of rice m^6^A writer genes

**Figure 2:**
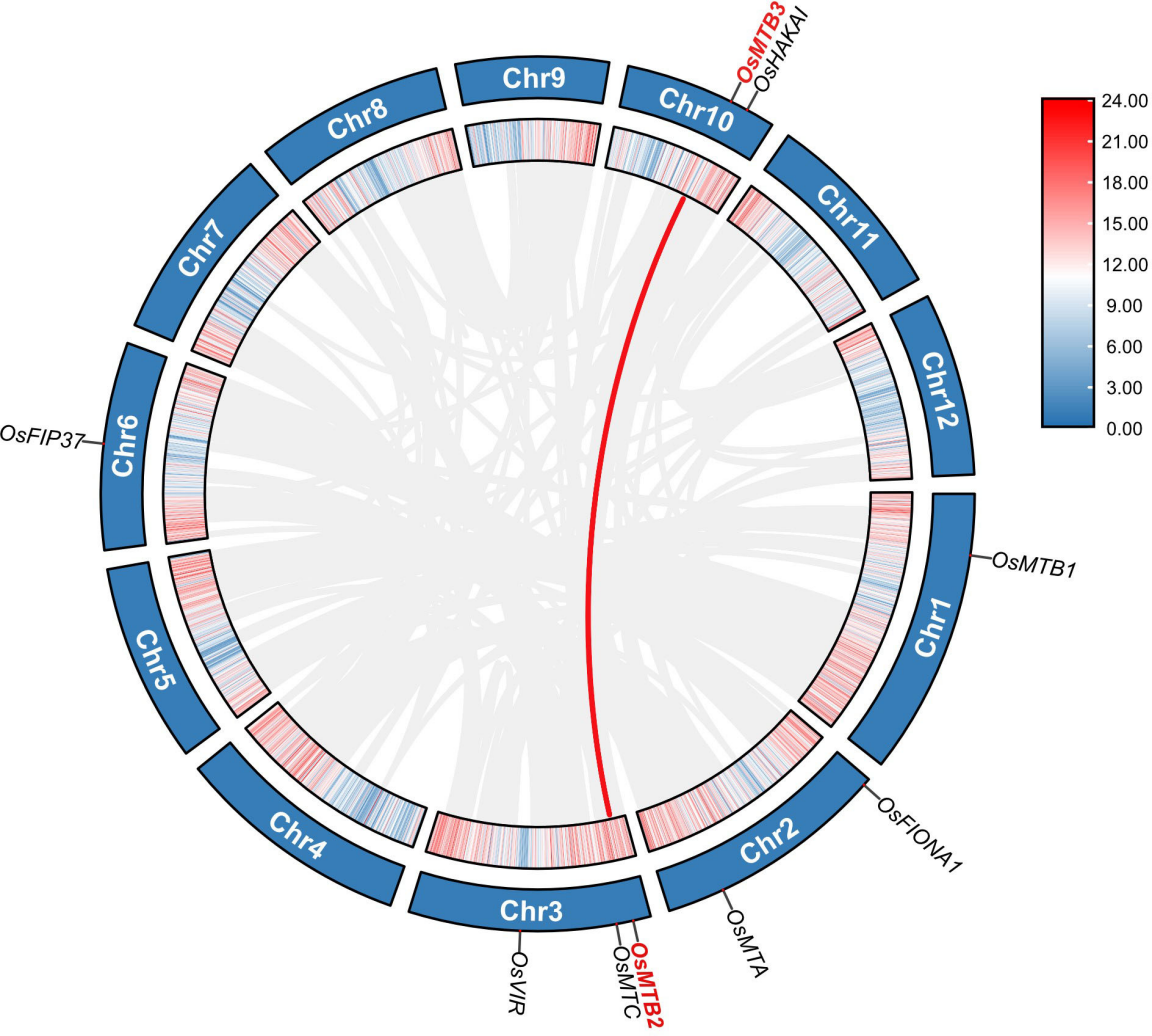
Collinearity analysis of the m^6^A writer genes in rice. The red lines represent collinear pairs of m^6^A writer genes, and the gray lines represent the covariance results of the rice genome. Concentric circles display rice chromosomes and gene density from outer to inner.

9 m^6^A methyltransferase genes were distributed on 5 chromosomes of *O. sativa* (**Figure 2**). Specifically, both chromosome 1 and chromosome 6 possessed one m^6^A writer gene, whereas chromosome 2 and chromosome 10 contained two m^6^A writers, respectively, while chromosome 3 had three of them. The collinearity analysis revealed no tandem duplication between the m^6^A writer genes. However, one pair resulting from fragment duplications (*OsMTB2*/*OsMTB3*) was observed, showing that this pair of genes may have originated from a common ancestral gene and undergone gene duplication events. Nevertheless, no synteny was observed among most m^6^A writer genes, indicating that the evolutionary history of m^6^A writer family is relatively complex, or that it has undergone extensive genetic rearrangements, losses, or duplications.

### 3.3 Predicted protein structures of m^6^A writers

**Figure 3:**
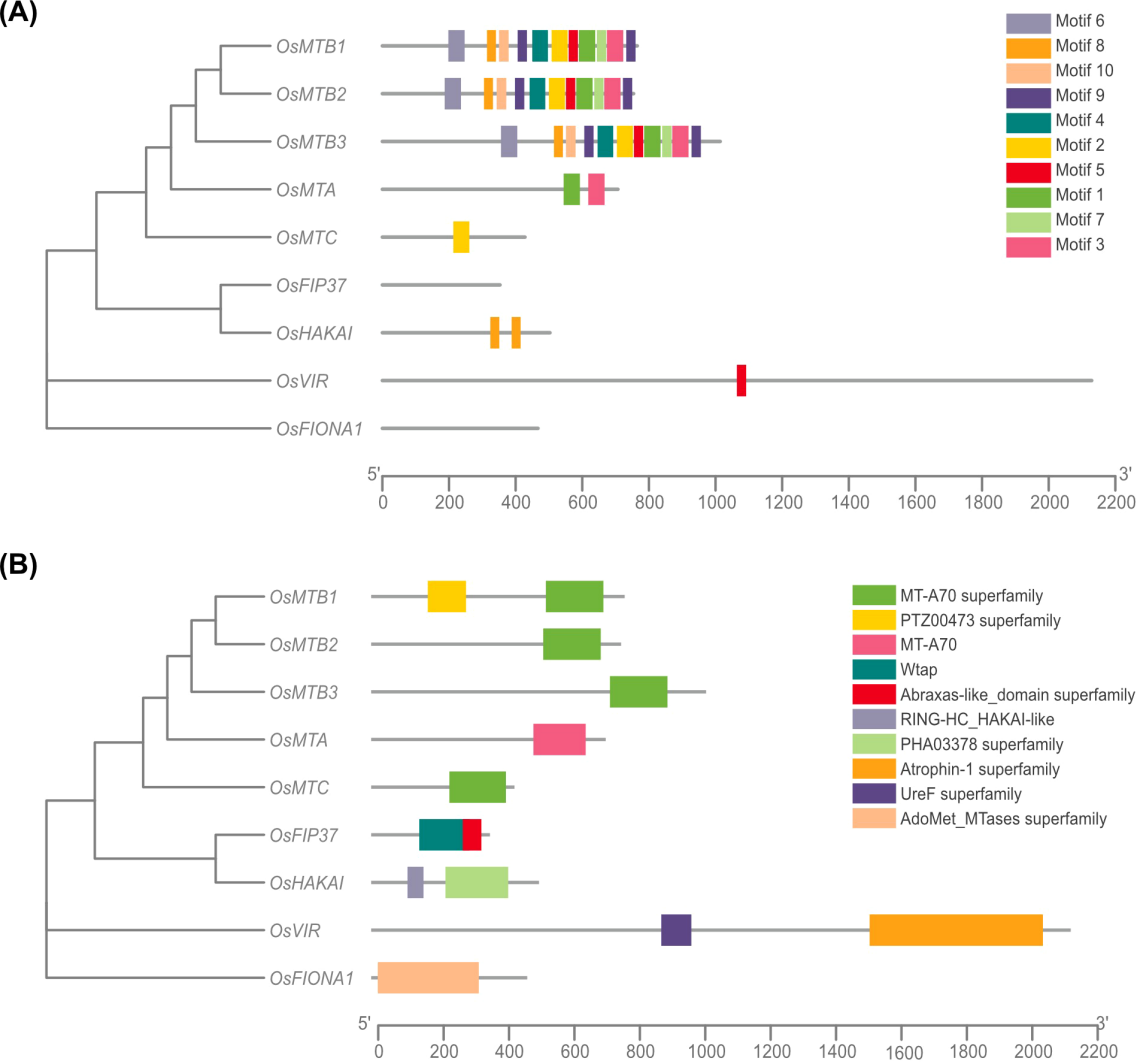
Motif composition and domain analyses of m^6^A writers in *O. sativa.* **(A)** Phylogenetic analysis coupled with motif distributions of m^6^A writer genes from *O. sativa*. **(B)** Phylogenetic analysis and conserved domain of m^6^A writer genes from *O. sativa*.

Analysis of conserved motifs is crucial for exploring the structural composition of rice m^6^A writer genes. In total, 10 distinct motifs were observed, among which motif 8 was commonly found in many members (**Figure 3A**). Furthermore, all of the motifs except for motif 5 and motif 8 exclusively existed in the MT-A70 members. Notably, *OsMTB1*, *OsMTB2*, and *OsMTB3* possessed the highest number of motifs, and their motif sequences were extremely similar in order, suggesting potential functional overlap. To investigate the potential functional diversity, the conserved domain was analyzed using CD-Search tool at NCBI and the results were visualized by TBtools. It is noted that the S-adenosylmethionine-binding domain MT-A70 could be found in *OsMTA*, while *OsMTB1*, *OsMTB2*, *OsMTB3*, and *OsMTC* possessed the MT-A70 superfamily. The PTZ00473 superfamily, which is not functionally characterized but is speculated to be a model that may span more than one domain, was observed in *OsMTB1*. Moreover, the component of m^6^A methyltransferase complex Wtap, as well as the Abraxas-like_domain superfamily (acting as a central scaffold protein that assembles the various components of the protein complex), were obtained in *OsFIP37*. *OsHAKAI* contained a RING-HC_HAKAI-like domain (possessing a Zn binding site and its dimerization forms a phosphotyrosine-binding pocket that recognizes specific phosphorylated tyrosine residues) and an unknown function PHA03378 superfamily. *OsVIR* consisted of UreF and Atrophin-1 superfamilies. UreF superfamily is proposed to regulate the activity of the relevant protein by eliminating the binding of nickel ions, while Atrophin-1 superfamily is involved in a progressive neurodegenerative disorder, dentatorubral-pallidoluysian atrophy. Last but not least, *OsFIONA1* comprised a S-adenosylmethionine-dependent methyltransferases (AdoMet_MTases) superfamily, which is involved in the installation of m^6^A for snRNA (**Figure 3B**).

**Figure 4:**
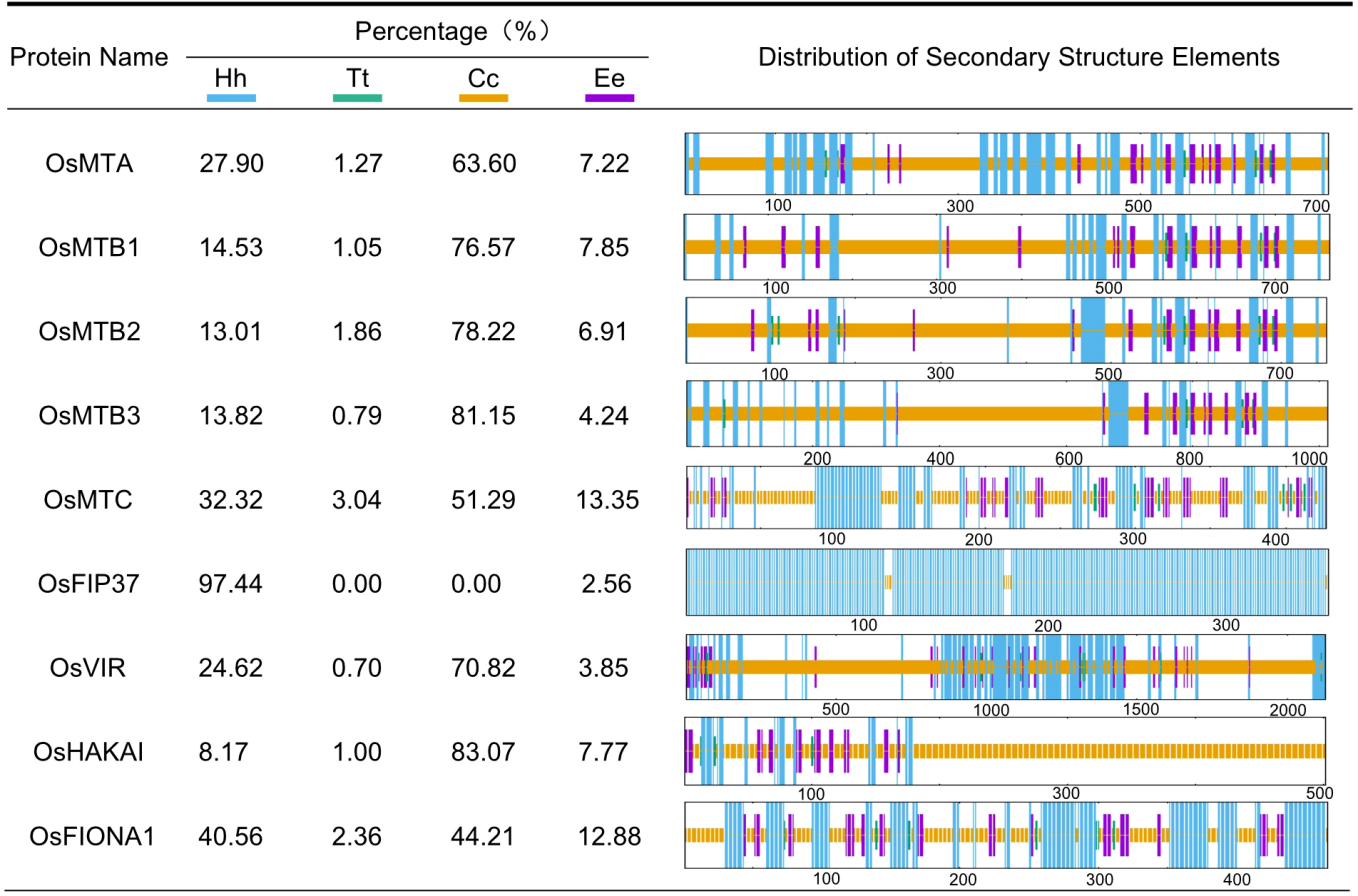
Secondary structure analysis of m^6^A writer proteins. The blue color represents alpha helix (Hh), the green color represents extended helix (Tt), the yellow color represents random coil (Cc) and the purple color represents extended strand (Ee).

**Figure 5:**
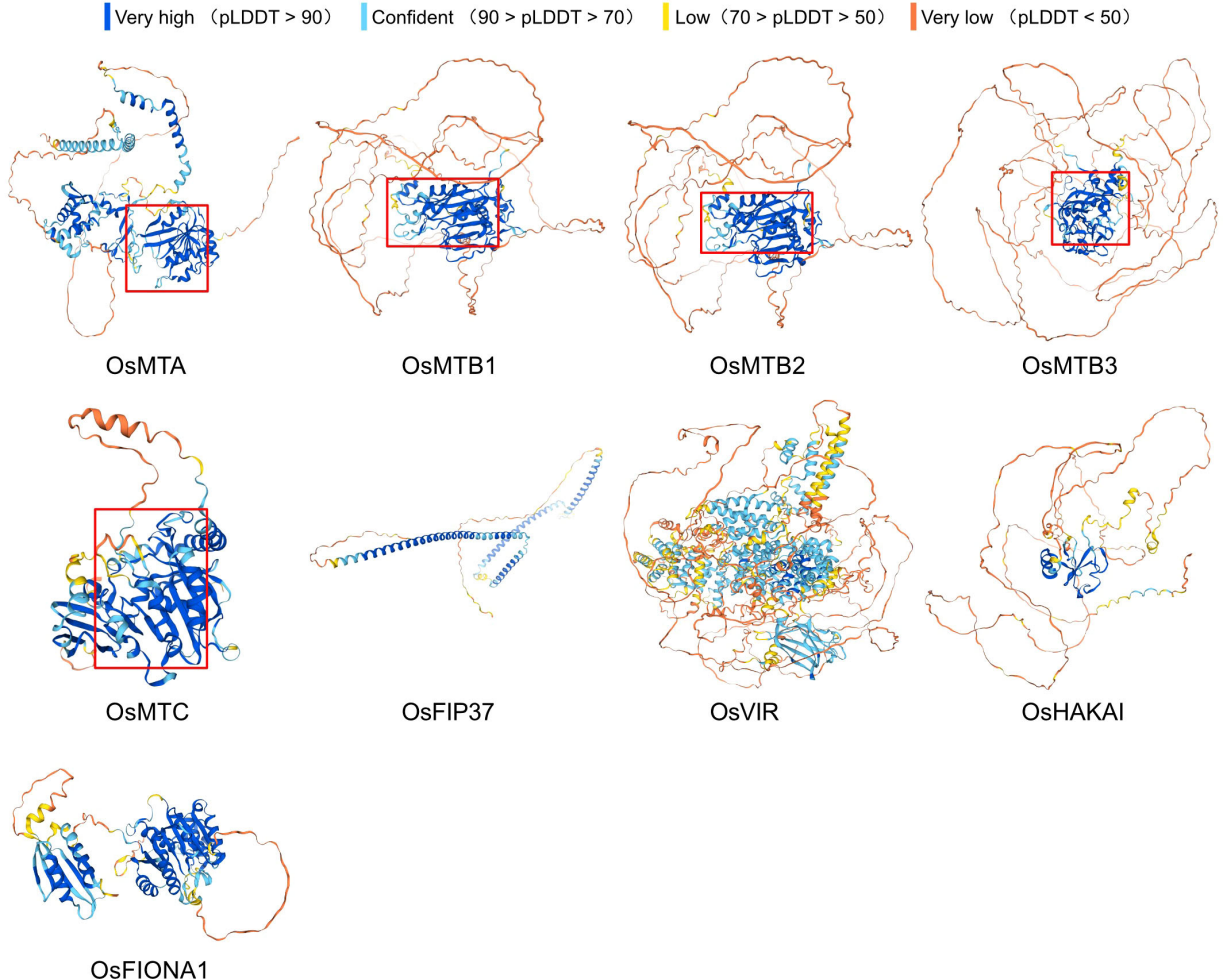
Tertiary structure prediction of m^6^A writers produced by SWISS-model. Alpha Fold v2 was selected as the running method. The active sites of the methyltransferases from the MT-A70 family are highlighted within the red box. The predicted local distance difference test (pLDDT) score (0-100) is displayed at the top of the figure.

To better understand the m^6^A writers, protein secondary structures were analyzed using the SOPMA online tool. OsFIP37 contains only alpha helices and extended strands, while all other m^6^A writers have four secondary structures: alpha helices, extended helices, random coils, and extended strands. Among these, random coils are the most abundant, followed by alpha helices, extended strands, and finally extended helices (**Figure 4**), indicating that, except for OsFIP37, random coils and alpha helices are the dominant conformations of m^6^A writer proteins. Furthermore, the prediction of tertiary structures revealed significant structural differences among most m^6^A writer proteins. Notably, OsMTB1, OsMTB2, and OsMTB3, all members of the MT-A70 subfamily, exhibited similar structures (**Figure 5**).

### 3.4 *Cis*-element analyses of m^6^A writer genes

**Figure 6:**
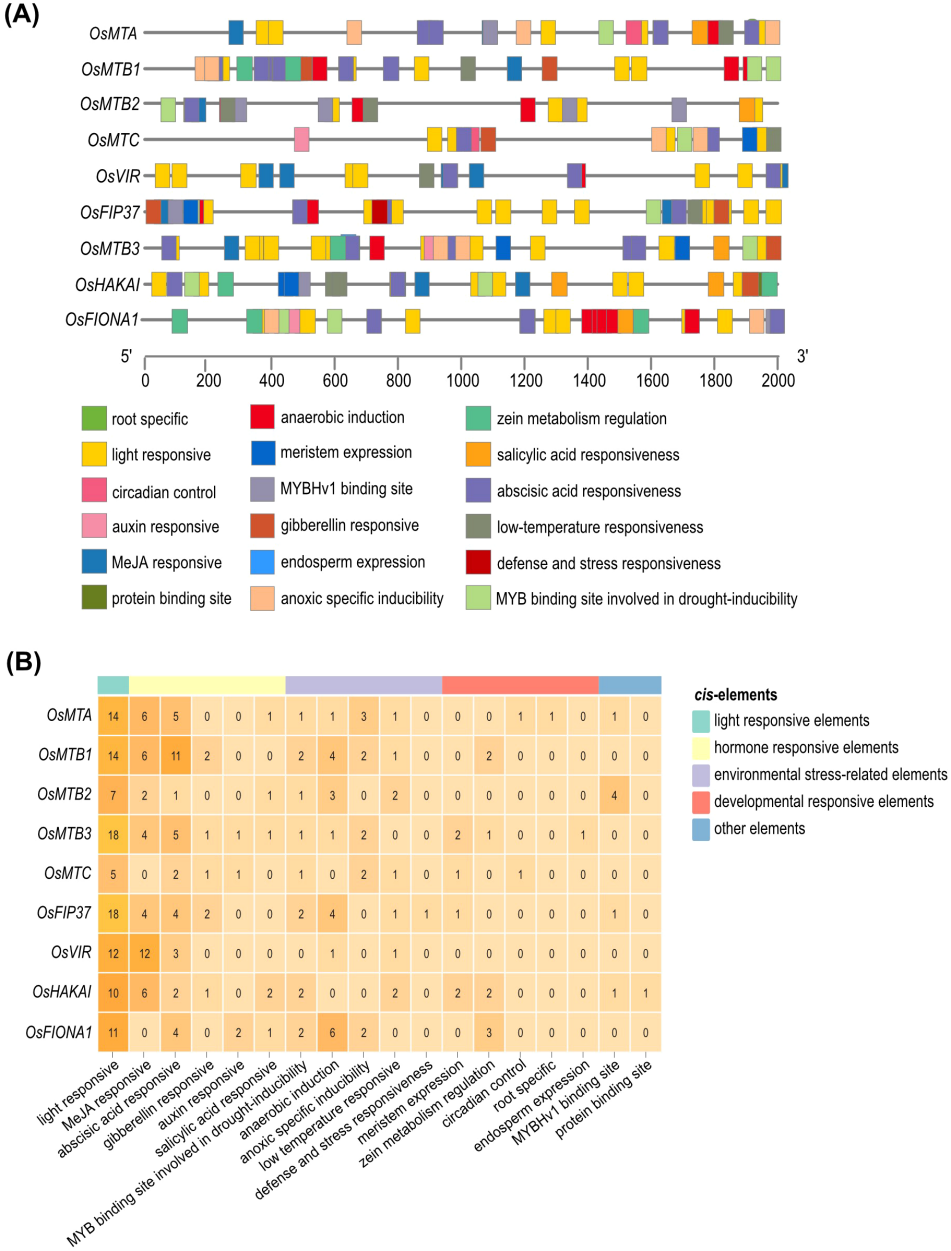
*Cis*-elements in the promoters of m^6^A writer genes. **(A)** The distribution of *cis*-elements in the promoters of the m^6^A writer genes. Different *cis-*elements were depicted in different colored boxes. **(B)** The number of each category of *cis-*element in m^6^A writer genes. *Cis-*elements were classified into those responsive to light, phytohormones, development, environmental stress, and other regulated categories.

In order to understand the regulatory activities of rice m^6^A writer genes, *cis*-elements were predicted within the 2000-bp promoter regions. PlantCARE, a plant promoter and *cis*-acting regulatory element database, was used for this analysis. The identified *cis*-acting elements encompassed 18 functional categories (**Figure 6A**), which could be grouped into five major classes: light-responsive elements, phytohormone-responsive elements, environmental stress-related elements, development-responsive elements, and other elements (**Figure 6B**). Among them, light-responsive elements and abscisic acid (ABA) responsive elements were identified in all promoters. For the phytohormone-responsive elements, those responding to methyl jasmonate (MeJA) were the most abundant, followed by those responding to ABA. In addition, the most frequent environmental stress-related elements were the MYB binding sites involved in drought inducibility and anaerobic induction. Last but not least, *cis*-elements related to the developmental response, like meristem expression, zein metabolism regulation, circadian control, root-specific, and endosperm expression, were also observed (**Figure 6B**).

### 3.5 Expression patterns of rice m^6^A writer genes in different tissues

**Figure 7:**
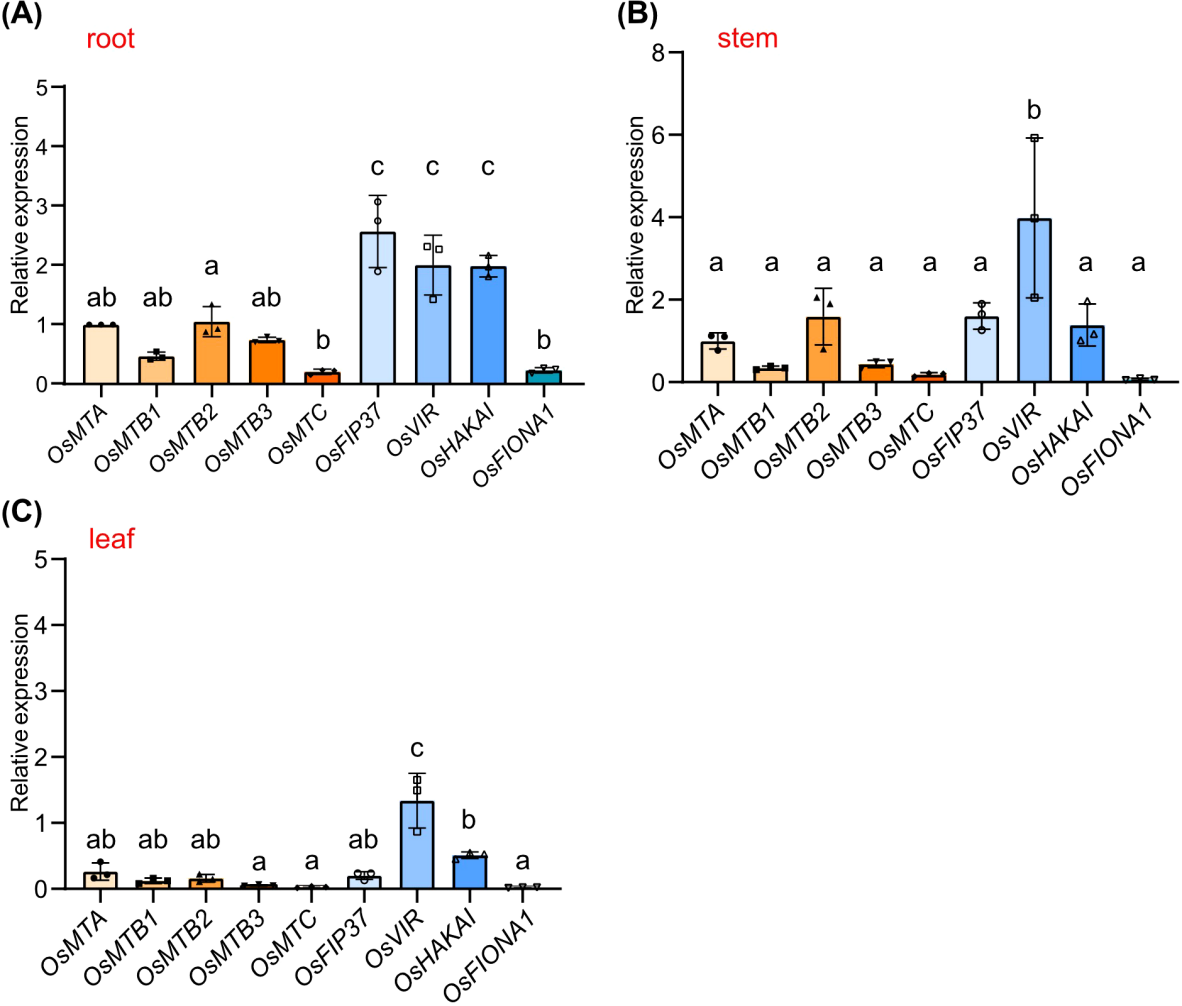
Expression analyses of the m^6^A writer genes in different tissues from 14-day-old rice seedlings, including root **(A)**, stem **(B)**, and leaf **(C)**, by qRT-PCR. *OsUBQ5* (Ubiquitin 5) was used as the internal control. Error bars indicate SD (n=3). Different letters indicate the significant difference at the P ≤ 0.05 level. One-way ANOVA multiple comparisons followed by the tukey test were employed for the statistical analysis.

The expression patterns of m^6^A writer genes were investigated by qRT-PCR in different tissues of rice seedlings, including roots, stems, and leaves (**Figure 7**). *OsMTA* expression level in roots was set as the calibration reference (expression level = 1) for normalization and comparison with the expression levels of others. It was found that *OsVIR* was highly expressed in roots, stems, and leaves, while *OsMTC* and *OsFIONA1* exhibited the lowest expression levels in leaves. In addition, *OsMTB2* was more highly expressed than *OsMTB1* and *OsMTB3* in all of the tissues. The expression levels of *OsMTA* and *OsMTC* were higher in roots and stems than in leaves. *OsFIP37*, *OsHAKAI*, and *OsFIONA1* were more highly expressed in roots than in stems and leaves, while *OsVIR* was more abundant in stems than in the other tissues (**Figure 7**).

### 3.6 Expression of m^6^A writer genes and m^6^A abundance in RNA response to cold stress

**Figure 8:**
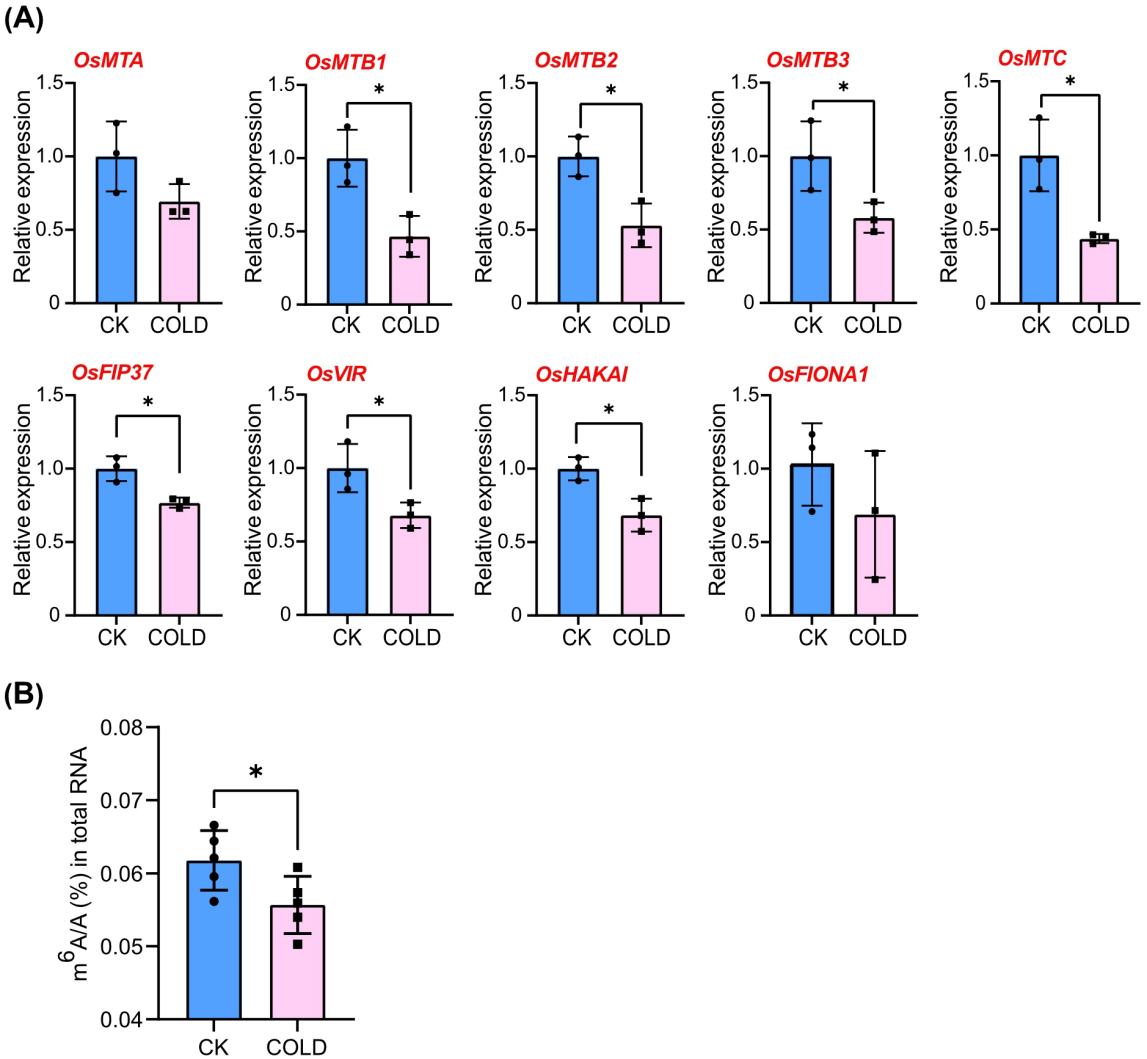
Relative expression levels of m^6^A writer genes, as well as the m^6^A abundance in total RNA under cold stress. **(A)** Relative expression levels of m^6^A writer genes using qRT-PCR. *OsUBQ5* (Ubiquitin 5) was used as the internal control. **(B)** The detection of m^6^A abundance in total RNA isolated from the seedlings under normal or low-temperature conditions was performed by LC-MS/MS. Error bars indicate SD (n=3 for qRT-PCR; n=5 for m^6^A abundance detection). Unpaired two-tailed t-test was employed as the statistical method. Asterisks indicate significant differences (*P ≤ 0.05).

To investigate the potential role of m^6^A writer genes, RT-qPCR was used to analyze the differences in transcription levels of 16-day-old rice seedlings under normal conditions or 6 hours of cold treatment. Interestingly, the expression level of all m^6^A writer genes showed a tendency to decrease under cold stress. Among them, *OsMTB1*, *MsMTB2*, *OsMTB3*, *OsMTC*, *OsFIP37*, *OsVIR*, and *OsHAKAI* were significantly downregulated (**Figure 8A**). Moreover, *OsMTC* exhibited the greatest downregulation at 56%, while *OsFIP37* showed the smallest downregulation at 23%, under cold stress. This result suggested that rice could respond to cold stress by decreasing the expression level of m^6^A writer genes. Due to the decreased expression of m^6^A writer genes, it could be speculated that the m^6^A abundance of rice RNA may also be reduced under cold treatment. Therefore, high performance liquid chromatography-tandem mass spectrometry (LC-MS/MS) was employed to detect the m^6^A abundance (m^6^A/A, the ratio of N6-methyladenosine to adenosine) in total RNAs of 16-day-old rice seedlings under normal conditions or 6 hours of cold treatment. As shown in **Figures 8B**, the level of m^6^A modification in total RNA from rice seedlings under cold stress was decreased, indicating that rice employed downregulation of RNA m^6^A abundance as a strategy in response to cold stress. However, the observed changes in the m^6^A/A ratio in samples collected under cold conditions may also be attributable to alterations in other RNA molecules, such as rRNA and tRNA, rather than effects on mRNA, which accounts for approximately 10% of total RNA.

**Figure 9:**
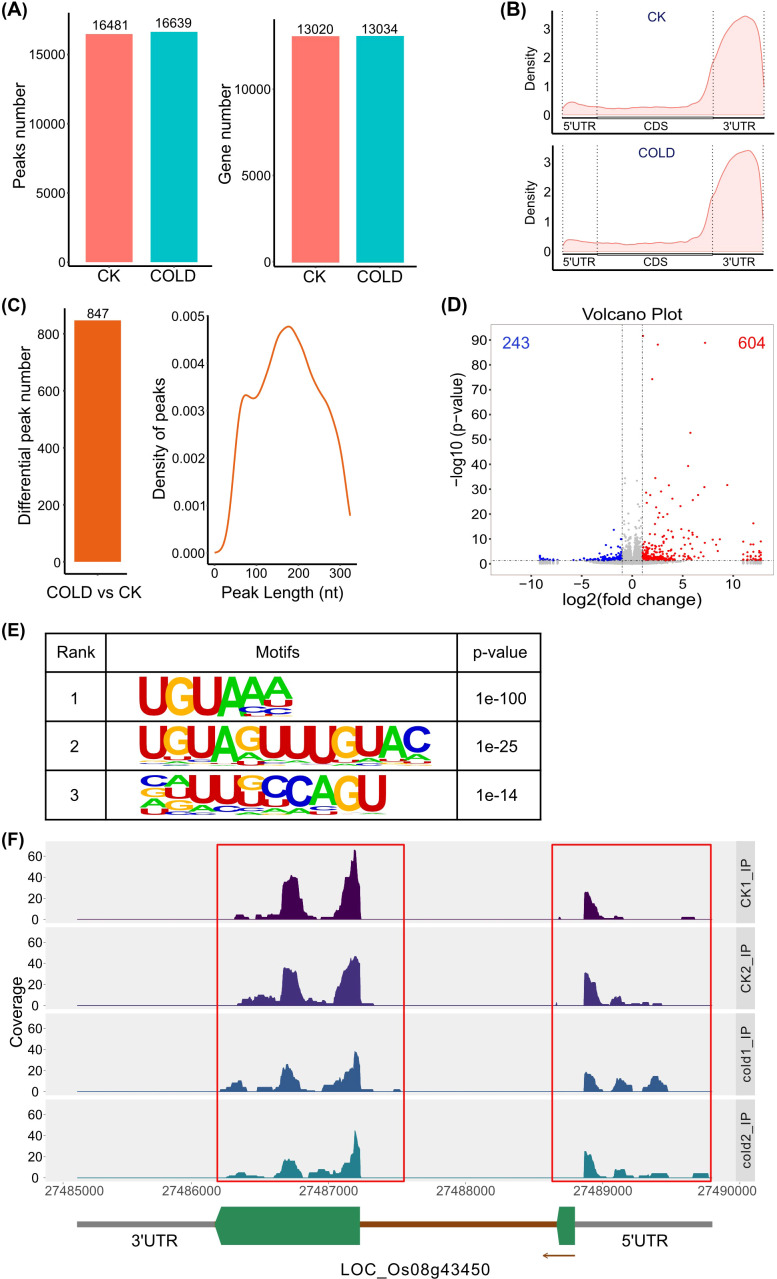
m^6^A methylation in the mRNA of rice seedlings under normal conditions (CK) and cold stress (COLD). **(A)** The number of m^6^A peaks identified at the transcriptional level in CK and COLD, along with their annotated gene counts. **(B)** Distribution of m^6^A peaks in functional regions of transcripts, including the 5’ untranslated region (5’UTR), coding sequence (CDS), and the 3’ untranslated region (3’UTR). **(C)** The number of m^6^A differential peaks between CK and COLD, along with their corresponding lengths. **(D)** Volcano plot of m^6^A differential peaks. Blue dots indicate downregulation of m^6^A abundance in differential peaks, while red dots indicate upregulation. Blue number represents the number of downregulated m^6^A peaks, and red number refers to the upregulated m^6^A peaks. **(E)** The top three enriched motifs of m^6^A me-RIP data, along with P values evaluated by HOMER. **(F)** Coverage of m^6^A peaks on LOC_Os08g43450. Gray pillars indicate untranslated regions, brown pillar represents the intron, and the green polygons refer to the exons. The m^6^A peaks are indicated in red boxes.

**Figure 10:**
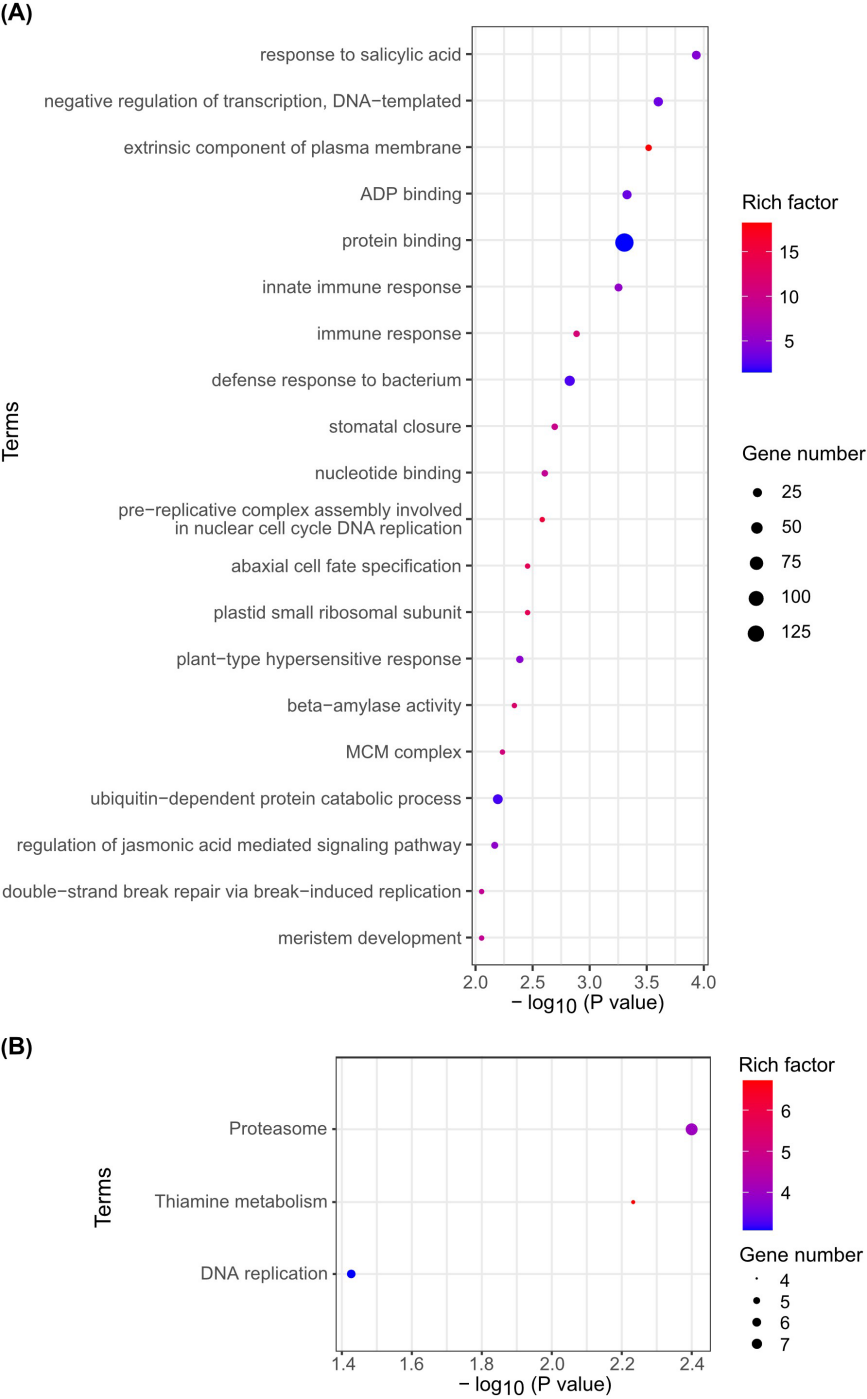
m^6^A differential peak annotated gene analyses of rice seedlings under normal conditions (CK) and cold stress (COLD). **(A)** Gene ontology (GO) enrichment analysis of m^6^A differential peak-associated genes. The size of the dots indicates the number of genes associated with differential m^6^A peaks within the GO Terms, while the darkness of the dots reflects the degree of enrichment by the rich factor. **(B)** Kyoto Encyclopedia of Genes and Genomes (KEGG) enrichment analysis of m^6^A differential peak-associated genes. The horizontal axis represents enrichment significance (expressed as -log10 (P value); higher values indicate stronger enrichment), while the vertical axis denotes the enriched KEGG pathway. Dot size corresponds to the number of genes associated with differential m^6^A peaks within that KEGG pathway, and dot intensity reflects the degree of enrichment by the rich factor.

Since m^6^A is one of the most abundant modifications in mRNA, methylated RNA immunoprecipitation with next generation sequencing (meRIP-seq) was performed to reveal the localization and overall status of m^6^A modification across the entire transcriptome of rice seedlings under normal conditions and cold stress. A total of 16481 and 16639 m^6^A peaks were observed in the mRNA from rice seedlings under normal conditions (CK) as well as cold stress (COLD), respectively. Moreover, 13020 genes were identified as containing m^6^A peaks in the CK group, while the cold treatment group identified 13034 genes (**Figure 9A**). Compared to the 5’ untranslated region (5’UTR) and coding sequence (CDS), the 3’ untranslated region (3’UTR) possessed the most abundant m^6^A modification peaks (**Figure 9B**). Besides, UGUAAA was identified as the most enriched motif containing m^6^A peaks (**Figure 9E**). There were 847 differentially m^6^A modified peaks at the transcriptional level, encompassing both up- and down-regulated peaks between cold-treated and control rice seedlings. The lengths of these differential peaks ranged from 20 to 320 nt, with the majority concentrated around 200 nt (**Figures 9C, D**). In addition, those differential m^6^A peaks were annotated to the genes involved in the influences of “response to salicylic acid”, “negative regulation of transcription, DNA-templated”, “extrinsic component of plasma membrane”, “ADP binding”, and “protein binding” via gene ontology (GO) enrichment analysis (**Figure 10A**), while Kyoto Encyclopedia of Genes and Genomes (KEGG) enrichment analysis revealed that the genes with differences in m^6^A abundance were participated in the biological processes like “proteasome”, “Thiamine metabolism”, and “DNA replication” (**Figure 10B**). Among them, the m^6^A level in the mRNA was decreased under cold stress of a putative MYB-like gene LOC_Os08g43450 (**Figure 9F**), which is presumably involved in the response to abiotic stresses in rice, as the transcription factors OsMYBs have been characterized to regulate adaptation to abiotic stresses. For instance, OsMYB2 was associated with the regulation of salt, cold, and drought tolerance (Yang et al., 2012), OsMYBS2 participated in the negative control of osmotic and drought stress (Chen et al., 2019), and OsMYB1R1 was involved in the negative regulation of drought resistance (Peng et al., 2023).

## 4 Discussion

N^6^-methyladenosine (m^6^A), a well-characterized RNA modification (particularly in mRNA), has been demonstrated to regulate multiple biological processes in plants, including yield development, nutritional growth, and stress adaptation (Chen et al., 2022). In this study, m^6^A writer genes were analyzed in various plant genomes by using the BLASTp method. The results showed that a total of 9 m^6^A writer genes were identified in *O. sativa*. The amino acid sequences of MT-A70 members in *O. sativa*, *A. thaliana*, *S. bicolor*, *Z. mays*, *P. vulgaris*, and *S. lycopersicum* were subsequently used to construct a phylogenetic tree, which indicated that despite evolutionary differences among those plants, they all retain the machinery for m^6^A modification. Despite a similar phylogenetic study has been reported previously (Yue et al., 2019), our study presents some unique data that is able to supplement prior research, such as the inclusion of additional species of *Phaseolus vulgaris*. In addition, the rice genome information referenced in this research is based on the japonica group possessing three MTB homologous genes (*OsMTB1*, *OsMTB2*, and *OsMTB3*), while Yue et al. selected indica group that contained only one MTB gene (*OiMTB*). Although most m^6^A writers were predicted to locate in the nucleus, while some of them exhibited distinct localization patterns, such as cytoplasm and plastid, which suggested that members of the m^6^A writer family in rice may undergo functional differentiation, performing different biological functions in various organelles. Despite significant variation in motif composition among all m^6^A writer candidates, the members of the MT-A70 family, *OsMTA*, *OsMTB1*, *OsMTB2*, *OsMTB3*, and *OsMTC*, exhibited similar motif to some degree. Furthermore, the high similarity in motif structure among *OsMTBs* (including *OsMTB1*, *OsMTB2*, and *OsMTB3*) not only suggested potential functional overlap but also indicated a degree of homology. *OsMTBs* possessed the most abundant and diverse motifs among m^6^A writer genes, demonstrating they may harbor unique functions and regulatory mechanisms. However, further researches are required to determine whether *OsMTBs* play the most critical role. Additionally, the analyses of secondary and tertiary structures (**Figures 4**, **5**) also reflected the structural similarity among MT-A70 family proteins. Comparatively, they shared a similar percentage of secondary structure elements, which had the proportions of random coil > alpha helix > extended strand > extended helix. Besides, OsFIP37 was almost entirely composed of alpha helix with a small percentage of extended strand. OsHAKAI had the highest percentage of random coil and the lowest percentage of alpha helix.

Gene duplication and collinearity analyses provided insights into the evolutionary dynamics of the rice m^6^A writer gene family. Among the nine identified m^6^A writer genes, one pair of segmental duplication events (*OsMTB2*/*OsMTB3*) was detected (**Figure 2**), suggesting that these two genes might originate from a common ancestral gene and undergo segmental duplication during genome evolution. Segmental duplication is a pivotal mechanism driving genome expansion and functional diversification, as it can increase gene dosage, generate novel gene functions, or form redundant functions, thereby enhancing an organism’s adaptability to changing environments. Even a single segmental duplication event could potentially contribute significantly to the functional diversity and adaptive capacity of the m^6^A writer gene family. However, the limited number of syntenic genes hindered the accurate determination of the expansion mode of this gene family. Gene family expansion is a complex process regulated by multiple factors, including selective pressure, gene recombination, and transposon activity. Thus, it is speculated that the rice m^6^A writer gene family may continue to undergo dynamic evolutionary changes. To fully elucidate the expansion patterns and evolutionary history of this gene family, additional studies incorporating more diverse rice accessions and closely related species are required, which will help clarify the evolutionary forces shaping the functional differentiation of these genes.

The analysis of *cis*-acting elements in the promoter regions of m^6^A writer genes sheds light on their potential regulatory roles in stress responses. The abundance of *cis*-elements responsive to light, methyl jasmonate (MeJA), and abscisic acid (ABA) indicated that m^6^A writer genes may be involved in ABA- and MeJA-mediated signaling pathways. Furthermore, 90% of the m^6^A writer genes contained *cis*-elements associated with drought, anaerobic, and low-temperature stresses (**Figure 6**), implying their potential participation in multiple abiotic stress responses. Previous studies have demonstrated that JA and ABA signaling pathways play crucial roles in plant cold resistance. Under cold stress, JA mitigates cold-induced photosynthetic inhibition in leaves through indirect mechanisms, whereas ABA primarily orchestrates root responses by inducing protective substances, especially dehydrins (Jarošová et al., 2024). Additionally, ABA could also act as a crucial component under cold stress, modulating the expression of Cold-Responsive (COR) genes via several transcription factors like bZIP, HOS members, and homo box (Guan et al., 2023).

In the model plant Arabidopsis, m^6^A has been characterized as a crucial RNA chemical modification, playing critical roles not only in growth and development but abiotic stress responses. For instance, among all mutants of the m^6^A writer component, the *vir* mutant with the most severe decrease in m^6^A levels exhibited a salt-sensitive phenotype. VIR-mediated m^6^A methylation was proven to regulate the homeostasis of reactive oxygen species by influencing the mRNA stability of NAC transcription factor (ATAF1), glutathione S-transferase U17 (GSTU17), and GIGANTEA (GI) (Hu et al., 2021). In rice, m^6^A modification has been reported to respond to salt and cadmium stresses (Li et al., 2025; Chen et al., 2023). Our study revealed that m^6^A modification is also involved in the response of rice to cold stress, not only in total RNA but also in mRNA. On the one hand, the decreased m^6^A level in total RNA of rice seedlings under cold stress, shown in **Figures 8B**, could possibly be a result of down-regulated expression level of m^6^A writer genes (**Figure 8A**). On the other hand, the decrease in m^6^A abundance could also be a consequence of a change in m^6^A levels in other types of RNA, such as rRNA and tRNA. Nearly a thousand differential peaks of m^6^A were identified in rice seedlings between normal conditions and cold treatment, providing a clue that rice may be able to alter the m^6^A levels in mRNA (**Figures 9**, **10**) by regulating the expression of m^6^A writer genes (**Figure 8A**), thereby modulating the post-transcriptional control of relevant genes to respond to cold stress. For instance, LOC_Os08g43450 is highlighted according to the m^6^A meRIP-seq result, not only because it exhibited a significant decrease in m^6^A levels within its mRNA, implying that its post-transcriptional regulation could be possibly influenced by m^6^A modification, but also it is predicted to be a MYB-like gene that likely plays a crucial role in abiotic stress responses, especially cold stress, making it of interest for future study. However, the biological functions of m^6^A modification regulating cold resistance in rice are still limited; further researches are required to elaborate the roles of RNA m^6^A modification in responding to cold stress.

## 5 Conclusion

In this study, the m^6^A writer gene family in rice was analyzed using bioinformatic methods. The results characterized these genes in terms of physicochemical properties, phylogenetic relationships, structural domain distributions, chromosomal localizations, and motif compositions. The *cis*-element analyses showed that MeJA- and ABA-related elements were fairly abundant. Given that prior studies have shown that ABA and MeJA play a significant role in cold resistance, it can be speculated that the expression of m^6^A writer genes could be influenced by the levels of MeJA and ABA. Additionally, the tissue-specific expression and response of the m^6^A writer genes to cold stress were investigated by qRT-PCR. Moreover, m^6^A abundance (ratio of m^6^A to A) detected by LC-MS/MS in total RNA of rice seedlings with cold treatment was decreased approximately from 0.062% to 0.057%. m^6^A meRIP-seq results demonstrated that the m^6^A modification was mainly distributed in 3’UTR, and 847 differential m^6^A peaks were identified in the mRNA of the rice seedlings between normal conditions and cold treatment. m^6^A levels were decreased in the transcripts of a series of genes, like LOC_Os08g43450, which is a putative transcription factor MYB-like gene with potential involvement in the response to cold stress, providing a clue that rice may respond to cold stress by regulating the m^6^A levels of certain genes, resulting in the post-regulation of cold-responsive genes. Nevertheless, further studies are required to reveal this molecular mechanism.

## Data Availability

The m^6^A meRIP-seq data have been uploaded to the NCBI database (accession number: PRJNA1417975).

## References

[B1] Achard P. Gong F. Cheminant S. Alioua M. Hedden P. Genschik P. (2008). The cold-inducible CBF1 factor-dependent signaling pathway modulates the accumulation of the growth-repressing DELLA proteins via its effect on gibberellin metabolism. Plant Cell 20, 2117–2129. doi: 10.1105/tpc.108.058941. PMID: 18757556 PMC2553604

[B2] Andrea C. Angana R. Elzbieta P. Sunandan M. Pietro B. Naeim S. . (2024). MODOMICS: A database of RNA modifications and related information. 2023 update. Nucleic Acids Res. 52, 239–244. doi: 10.1093/nar/gkad1083. PMID: 38015436 PMC10767930

[B3] Bodi Z. Zhong S. Mehra S. Song J. Graham N. Li H. . (2012). Adenosine methylation in Arabidopsis mRNA is associated with the 3′ end and reduced levels cause developmental defects. Front. Plant 3. doi: 10.3389/fpls.2012.00048. PMID: 22639649 PMC3355605

[B4] Cai J. Hu J. Xu T. Kang H. (2024). FIONA1-mediated mRNA m^6^A methylation regulates the response of Arabidopsis to salt stress. Plant Cell Environ. 47, 900–912. doi: 10.1111/pce.14807. PMID: 38193282

[B5] Chen D. Fu L. Su T. Xiong J. Chen Y. Shen Q. . (2022). N6-methyladenosine methylation analysis reveals transcriptome-wide expression response to salt stress in rice roots. Environ. Exp. Bot. 201, 104945. doi: 10.1016/j.envexpbot.2022.104945. PMID: 41878731

[B6] Chen J. Cao H. Chen D. Kuang L. H. Wu D. Z. (2023). Transcriptome-wide analysis of m6A methylation reveals genetic responses to cadmium stress at germination stage in rice. Environ. Exp. Bot. 205, 105130. doi: 10.1016/j.envexpbot.2022.105130. PMID: 41878731

[B7] Chen Y. Ho T. Liu L. Lee D. Lee C. Chen Y. . (2019). Sugar starvation-regulated MYBS2 and 14-3–3 protein interactions enhance plant growth, stress tolerance, and grain weight in rice. Proc. Natl. Acad. Sci. 43, 21925–21935. doi: 10.1073/pnas.1904818116. PMID: 31594849 PMC6815185

[B8] Fujino K. Sekiguchi H. Matsuda Y. Sugimoto K. Ono K. Yano M. (2008). Molecular identification of a major quantitative trait locus, qLTG3-1, controlling low-temperature germinability in rice. Proc. Natl. Acad. Sci. U.S.A. 105, 12623–12628. doi: 10.1073/pnas.0805303105. PMID: 18719107 PMC2527961

[B9] Guan Y. L. Hwarari D. Korboe H. M. Ahmad B. Cao Y. W. Movahedi A. . (2023). Low temperature stress-induced perception and molecular signaling pathways in plants. Environ. Exp. Bot. 207, 105190. doi: 10.1016/j.envexpbot.2022.105190. PMID: 41878731

[B10] Han B. Wei S. Li F. Zhang J. Li Z. Gao X. (2021). Decoding m^6^A mRNA methylation by reader proteins in cancer. Cancer Lett. 518, 256–265. doi: 10.1016/j.canlet.2021.07.047. PMID: 34339799

[B11] Hu J. Cai J. Park S. J. Lee K. Li Y. Chen Y. . (2021). N6-methyladenosine mRNA methylation is important for salt stress tolerance in Arabidopsis. Plant J. 106, 1759–1775. doi: 10.1111/tpj.15270. PMID: 33843075

[B12] Huang Y. Zheng P. Liu X. Chen H. Tu J. (2021). OseIF3h regulates plant growth and pollen development at translational level presumably through interaction with OsMTA2. Plants 30, 1101. doi: 10.3390/plants10061101. PMID: 34070794 PMC8228589

[B13] Jarošová J. Prerostova S. Černý M. Dobrev P. Gaudinova A. Knirsch V. . (2024). Hormonal responses of rice to organ-targeted cold stress. Environ. Exp. Bot. 222, 105739. doi: 10.1016/j.envexpbot.2024.105739. PMID: 41878731

[B14] Jeon J. Kim N. Y. Kim S. Kang N. Y. Kim J. (2010). A subset of cytokinin two-component signaling system plays a role in cold temperature stress response in Arabidopsis. J. Biol. Chem. 285, 23371–23386. doi: 10.1074/jbc.M109.096644. PMID: 20463025 PMC2906329

[B15] Jia G. Fu Y. He C. (2013). Reversible RNA adenosine methylation in biological regulation. Trends Genet. 29, 108–115. doi: 10.1016/j.tig.2012.11.003. PMID: 23218460 PMC3558665

[B16] Kim S. I. Andaya V. C. Tai T. H. (2011). Cold sensitivity in rice (Oryza sativa L.) is strongly correlated with a naturally occurring I99V mutation in the multifunctional glutathione transferase isoenzyme GSTZ2. Biochem. J. 435, 373–380. doi: 10.1042/BJ20101610. PMID: 21281270

[B17] Li Y. Yin M. Wang J. Zhao X. Xu J. Wang W. . (2025). Epitranscriptome profiles reveal participation of the RNA methyltransferase gene OsMTA1 in rice seed germination and salt stress response. BMC Plant Biol. 25, 115. doi: 10.1186/s12870-025-06134-4. PMID: 39865266 PMC11771074

[B18] Liu P. Liu H. Zhao J. Yang T. Guo S. Chang L. . (2024). Genome-wide identification and functional analysis of mRNA m^6^A writers in soybean under abiotic stress. Front. Plant Sci. 15. doi: 10.3389/fpls.2024.1446591. PMID: 39055358 PMC11269220

[B19] Luo Q. Mo J. Chen H. Hu Z. Wang B. Wu J. . (2022). Structural insights into molecular mechanism for N6-adenosine methylation by MT-A70 family methyltransferase METTL4. Nat. Commun. 13, 5636. doi: 10.1038/s41467-022-33277-x. PMID: 36163360 PMC9512776

[B22] Ma Y. Dai X. Xu Y. Luo W. Zheng X. Zeng D. . (2015). COLD1 confers chilling tolerance in rice. Cell. 160, 1209–1221. doi: 10.1016/j.cell.2015.01.046. PMID: 25728666

[B21] Ma K. Han J. Zhang Z. Li H. Zhao Y. Zhu Q. . (2021). OsEDM2L mediates m^6^A of EAT1 transcript for proper alternative splicing and polyadenylation regulating rice tapetal degradation. J. Integr. Plant Biol. 63, 1982–1994. doi: 10.1111/jipb.13167. PMID: 34449974

[B20] Ma H. Zhu X. Cao T. Li S. Guo E. Shi Y. . (2023). Responses of rice cultivars with different cold tolerance to chilling in booting and flowering stages: An experiment in Northeast China. J. Agro. Crop Sci. 209, 864–875. doi: 10.1111/jac.12663. PMID: 41875165

[B23] Mao D. Xin Y. Tan Y. Hu X. Bai J. Liu Z. Y. . (2019). Natural variation in the HAN1 gene confers chilling tolerance in rice and allowed adaptation to a temperate climate. Proc. Natl. Acad. Sci. U.S.A. 116, 3494–3501. doi: 10.1073/pnas.1819769116. PMID: 30808744 PMC6397538

[B24] Maruyama K. Urano K. Yoshiwara K. Morishita Y. Sakurai N. Suzuki H. . (2014). Integrated analysis of the effects of cold and dehydration on rice metabolites, phytohormones, and gene transcripts. Plant Physiol. 164, 1759–1771. doi: 10.1104/pp.113.231720. PMID: 24515831 PMC3982739

[B25] Peng Y. Tang N. Zou J. Ran J. Chen X. (2023). Rice MYB transcription factor OsMYB1R1 negatively regulates drought resistance. Plant Growth Regul. 99, 515–525. doi: 10.1007/s10725-022-00922-w. PMID: 41878318

[B26] Rahemi M. Yazdani F. Sedaghat S. (2016). Evaluation of freezing tolerance in olive cultivars by stomatal density and freezing stress. Int. J. Hortic. Sci. Technol. 2, 145–153. doi: 10.22059/IJHST.2016.62914

[B27] Rahman A. (2013). Auxin: a regulator of cold stress response. Physiol. Plantarum. 147, 28–35. doi: 10.1111/j.1399-3054.2012.01617.x. PMID: 22435366

[B28] Růžička K. Zhang M. Campilho A. Bodi Z. Kashif M. Saleh M. . (2017). Identification of factors required for m^6^A mRNA methylation in Arabidopsis reveals a role for the conserved E3 ubiquitin ligase HAKAI. New Phytol. 215, 157–172. doi: 10.1111/nph.14586. PMID: 28503769 PMC5488176

[B29] Saito K. Hayano-Saito Y. Kuroki M. Sato Y. (2010). Map-based cloning of the rice cold tolerance gene Ctb1. Plant Sci. 179, 97–102. doi: 10.1016/j.plantsci.2010.04.004. PMID: 41878731

[B30] Seoa J. Yi G. Lee J. G. Choi J. H. Lee E. J. (2020). Seed browning in pepper (Capsicum annuum L.) fruit during cold storage is inhibited by methyl jasmonate or induced by methyl salicylate. Postharvest. Biol. Tec. 166, 111210. doi: 10.1016/j.postharvbio.2020.111210. PMID: 41878731

[B31] Shen L. Liang Z. Gu X. Chen Y. Teo Z. W. Hou X. . (2016). N (6)-methyladenosine RNA modification regulates shoot stem cell fate in Arabidopsis. Dev. Cell 38, 186–200. doi: 10.1016/j.devcel.2016.06.008. PMID: 27396363 PMC6364302

[B33] Shi Y. Tian S. Hou L. Huang X. Zhang X. Guo H. . (2012). Ethylene signaling negatively regulates freezing tolerance by repressing expression of CBF and type-A ARR genes in Arabidopsis. Plant Cell 24, 2578–2595. doi: 10.1105/tpc.112.098640. PMID: 22706288 PMC3406918

[B32] Shi H. Wei J. He C. (2019). Where, when, and how: Context-dependent functions of RNA methylation writers, readers, and erasers. Mol. Cell 74, 640–650. doi: 10.1016/j.molcel.2019.04.025. PMID: 31100245 PMC6527355

[B35] Wang Z. Sun J. Zu X. Gong J. Deng H. Hang R. . (2022). Pseudouridylation of chloroplast ribosomal RNA contributes to low temperature acclimation in rice. New Phytol. 236, 1708–1720. doi: 10.1111/nph.18479. PMID: 36093745

[B34] Wang C. Yang J. Song P. Zhang W. Lu Q. Yu Q. . (2022). FIONA1 is an RNA N^6^-methyladenosine methyltransferase affecting Arabidopsis photomorphogenesis and flowering. Genome Biol. 23, 40. doi: 10.1186/s13059-022-02612-2. PMID: 35101091 PMC8802475

[B36] Xin Z. Browse J. (1998). Eskimo1 mutants of Arabidopsis are constitutionally freezing-tolerant. Proc. Natl. Acad. Sci. U.S.A. 95, 7799–7804. doi: 10.1073/PNAS.95.13.7799. PMID: 9636231 PMC22762

[B37] Xu Y. Wang R. Wang Y. Zhang L. Yao S. (2020). A point mutation in LTT1 enhances cold tolerance at the booting stage in rice. Plant Cell Environ. 43, 992–1007. doi: 10.1111/pce.13717. PMID: 31922260 PMC7154693

[B38] Yang A. Dai X. Zhang W. (2012). A R2R3-type MYB gene, OsMYB2, is involved in salt, cold, and dehydration tolerance in rice. J. Exp. Bot. 63, 2541–2556. doi: 10.1093/jxb/err431. PMID: 22301384 PMC3346221

[B39] Yue H. Nie X. Yan Z. Weining S. (2019). N6-methyladenosine regulatory machinery in plants: composition, function and evolution. Plant Biotechnol. J. 17, 1194–1208. doi: 10.1111/pbi.13149. PMID: 31070865 PMC6576107

[B41] Zhang Z. Huang R. (2010). Enhanced tolerance to freezing in tobacco and tomato overexpressing transcription factor TERF2/LeERF2 is modulated by ethylene biosynthesis. Plant Mol. Biol. 73, 241–249. doi: 10.1007/s11103-010-9609-4. PMID: 20135196

[B42] Zhang Z. Li J. Pan Y. Li J. Zhou L. Shi H. . (2017). Natural variation in CTB4a enhances rice adaptation to cold habitats. Nat. Commun. 8, 14788. doi: 10.1038/ncomms14788. PMID: 28332574 PMC5376651

[B40] Zhang S. Zheng J. Liu B. Peng S. Leung H. Zhao J. . (2014). Identification of QTLs for cold tolerance at seedling stage in rice (Oryza sativa L.) using two distinct methods of cold treatment. Euphytica. 195, 95–104. doi: 10.1007/s10681-013-0977-0. PMID: 41878318

[B43] Zheng H. Sun X. Li J. Song Y. Song J. Wang F. . (2021). Analysis of N6-methyladenosine reveals a new important mechanism regulating the salt tolerance of sweet sorghum. Plant Sci. 304, 110801. doi: 10.1016/j.plantsci.2020.110801. PMID: 33568300

[B44] Zhou Y. Kong Y. Fan W. Tao T. Xiao Q. Li N. . (2020). Principles of RNA methylation and their implications for biology and medicine. Biomed. Pharmacother. 131, 110731. doi: 10.1016/j.biopha.2020.110731. PMID: 32920520

