# Supplementary material for: Genome-wide identification of m6A methyltransferase genes and m6A modification participates in the response to cold stress in rice
Source: Front Plant Sci. 2026 Apr 13;17:1804596. doi: 10.3389/fpls.2026.1804596 (PMC13111082; doi:10.3389/fpls.2026.1804596)
Supplement: Supplementary file 1 [file Table1.docx]

| Gene name | Primer name | sequences |
| --- | --- | --- |
| *OsUBQ5* | P78 | \| CTGCTGCTGTTCTTGGGTTCA \| \| --- \| |
|  | P79 | TCATTATAGTTCTTCCATGCTGCTC |
| *OsMTA* | P226 | \| GAAGGAGACGGCTGTGGCA \| \| --- \| |
|  | P227 | \| AATACGGCGGAAATGGACC \| \| --- \| |
| *OsMTB1* | P230 | \| CTGTTCCAACCAACCTTCCTAT \| \| --- \| |
|  | P231 | \| ACCCACTGCCTGTTTGATTTA \| \| --- \| |
| *OsMTB2* | P234 | \| CACCTAAACCAAATCGGCTCT \| \| --- \| |
|  | P235 | \| AATCATTCTGCTCACCCCTTG \| \| --- \| |
| *OsMTB3* | P240 | \| TAGCCGATGAACCAACCGAT \| \| --- \| |
|  | P241 | \| TCCCAAGAGTAAGCCAACCC \| \| --- \| |
| *OsMTC* | P242 | \| CCCTACACTTCCCAACAGACAT \| \| --- \| |
|  | P243 | \| ACTCCCCAAGCAGGAAATAAC \| \| --- \| |
| *OsFIP37* | P246 | \| GCTGCTGTCAATTTTACTCCATC \| \| --- \| |
|  | P247 | \| AGAAGCCATCGCCCCAATC \| \| --- \| |
| *OsVIR* | P252 | \| TGTCACCCCTTCCTCTACGG \| \| --- \| |
|  | P253 | \| AACACCAGACCCTCCTTGATTAT \| \| --- \| |
| *OsHAKAI* | P254 | \| TGGTAGTCAAGACAAACAGCAAA \| \| --- \| |
|  | P255 | \| GGAAAGGGGCACTGAAGAAC \| \| --- \| |
| *OsFIONA1* | P340 | CTAGGCTGGAGCTTTGTTGGA |
|  | P341 | TGCTAGCTGTGGATTGCTCT |
