# Supplementary material for: Genome-wide identification of m6A methyltransferase genes and m6A modification participates in the response to cold stress in rice
Source: Front Plant Sci. 2026 Apr 13;17:1804596. doi: 10.3389/fpls.2026.1804596 (PMC13111082; doi:10.3389/fpls.2026.1804596)
Supplement: Supplementary file 2 [file Table2.docx]

| Family | Organism | Gene name | Gene ID  (Phytozome) | Amino  acid  length | Isoelectric  point | Molecular  weight  (kDa) | Subcellular  localization  prediction | Orthologous  gene ID in A. thaliana |  |
| --- | --- | --- | --- | --- | --- | --- | --- | --- | --- |
| MTA-70 | *Oryza sativa* | *OsMTA* | LOC_Os02g45110 | 706 | 6.76 | 77.80 | nucleus.  cytoplasm | AT4G10760 |  |
|  | *Sorghum bicolor* | *SbMTA* | Sobic.004G285000 | 706 | 6.44 | 77.78 | cytoplasm | AT4G10760 |  |
|  | *Zea mays* | *ZmMTA* | Zm00001d017566 | 704 | 6.93 | 77.86 | cytoplasm | AT4G10760 |  |
|  | *Arabidopsis thaliana* | *AtMTA* | AT4G10760 | 685 | 6.17 | 76.64 | cytoplasm | AT4G10760 |  |
|  | *Phaseolus vulgaris* | *PvMTA* | Phvul.010G102500 | 761 | 6.01 | 84.32 | chloroplast | AT4G10760 |  |
|  | *Solanum lycopersicum* | *SlMTA* | Solyc08g066730 | 739 | 6.43 | 81.47 | chloroplast | AT4G10760 |  |
|  | *Oryza sativa* | *OsMTB1* | LOC_Os01g16180 | 764 | 6.75 | 85.30 | nucleus | AT4G09980 |  |
|  | *Oryza sativa* | *OsMTB2* | LOC_Os03g05420 | 753 | 6.75 | 83.59 | nucleus | AT4G09980 |  |
|  | *Oryza sativa* | *OsMTB3* | LOC_Os10g31030 | 1013 | 6.17 | 113.61 | nucleus | AT4G09980 |  |
|  | *Sorghum bicolor* | *SbMTB* | Sobic.001G503900 | 804 | 6.54 | 90.61 | nucleus | AT4G09980 |  |
|  | *Zea mays* | *ZmMTB* | Zm00001d027671 | 808 | 6.71 | 91.28 | nucleus | AT4G09980 |  |
|  | *Arabidopsis thaliana* | *AtMTB* | AT4G09980 | 963 | 8.47 | 106.36 | nucleus | AT4G09980 |  |
|  | *Phaseolus vulgaris* | *PvMTB* | Phvul.007G073300 | 1086 | 6.86 | 120.67 | nucleus | AT4G09980 |  |
|  | *Solanum lycopersicum* | *SlMTB1* | Solyc05g056220 | 1091 | 6.39 | 121.98 | nucleus | AT4G09980 |  |
|  | *Solanum lycopersicum* | *SlMTB2* | Solyc05g056210 | 1094 | 6.34 | 122.63 | nucleus | AT4G09980 |  |
|  | *Oryza sativa* | *OsMTC* | LOC_Os03g10224 | 427 | 8.49 | 49.39 | nucleus | AT1G19340 |  |
|  | *Sorghum bicolor* | *SbMTC* | Sobic.001G468200 | 416 | 7.19 | 48.06 | cytoplasm | AT1G19340 |  |
|  | *Zea mays* | *ZmMTC* | Zm00001d028128 | 697 | 9.35 | 78.96 | nucleus | AT1G19340 |  |
|  | *Arabidopsis thaliana* | *AtMTC* | AT1G19340 | 414 | 6.08 | 47.89 | nucleus | AT1G19340 |  |
|  | *Phaseolus vulgaris* | *PvMTC* | Phvul.001G016200 | 427 | 8.04 | 48.64 | nucleus | AT1G19340 |  |
|  | *Solanum lycopersicum* | *SlMTC* | Solyc04g079950 | 419 | 6.73 | 48.10 | nucleus | AT1G19340 |  |
| WTAP | *Oryza sativa* | *OsFIP37* | LOC_Os06g27970 | 352 | 5.11 | 39.36 | nucleoplam | AT3G54170 |  |
|  | *Sorghum bicolor* | *SbFIP37* | Sobic.004G033100 | 369 | 5.01 | 41.05 | nucleus | AT3G54170 |  |
|  | *Zea mays* | *ZmFIP37* | Zm00001d015194 | 370 | 4.91 | 40.82 | nucleus | AT3G54170 |  |
|  | *Arabidopsis thaliana* | *AtFIP37* | AT3G54170 | 330 | 4.92 | 37.21 | nucleus | AT3G54170 |  |
|  | *Phaseolus vulgaris* | *PvFIP37* | Phvul.002G107400 | 337 | 5.41 | 38.15 | nucleus | AT3G54170 |  |
|  | *Solanum lycopersicum* | *SlFIP37* | Solyc03g112520 | 342 | 4.86 | 38.64 | cytoplasm | AT3G54170 |  |
| VIR | *Oryza sativa* | *OsVIR* | LOC_Os03g35340 | 2128 | 5.16 | 233.75 | plastid | AT3G05680 |  |
|  | *Sorghum bicolor* | *SbVIR* | Sobic.001G191700 | 2212 | 5.29 | 243.53 | plastid | AT3G05680 |  |
|  | *Zea mays* | *ZmVIR* | Zm00001d013829 | 2219 | 5.35 | 244.89 | plastid | AT3G05680 |  |
|  | *Arabidopsis thaliana* | *AtVIR* | AT3G05680 | 2152 | 5.40 | 235.76 | plastid | AT3G05680 |  |
|  | *Phaseolus vulgaris* | *PvVIR* | Phvul.007G267500 | 2188 | 5.35 | 240.79 | plastid | AT3G05680 |  |
|  | *Solanum lycopersicum* | *SlVIR* | Solyc03g020020 | 2196 | 5.49 | 240.80 | plastid | AT3G05680 |  |
| HAKAI | *Oryza sativa* | *OsHAKAI* | LOC_Os10g35190 | 502 | 6.70 | 53.12 | nucleus | AT5G01160 |  |
|  | *Sorghum bicolor* | *SbHAKAI* | Sobic.001G207500 | 480 | 6.42 | 51.56 | nucleus | AT5G01160 |  |
|  | *Zea mays* | *ZmHAKAI1* | Zm00001d032600 | 508 | 7.15 | 54.68 | nucleus | AT5G01160 |  |
|  | *Zea mays* | *ZmHAKAI2* | Zm00001d013962 | 500 | 7.13 | 54.02 | nucleus | AT5G01160 |  |
|  | *Arabidopsis thaliana* | *AtHAKAI* | AT5G01160 | 360 | 6.45 | 39.77 | nucleus | AT5G01160 |  |
|  | *Phaseolus vulgaris* | *PvHAKAI* | Phvul.008G108800 | 437 | 6.27 | 47.77 | nucleus | AT5G01160 |  |
|  | *Solanum lycopersicum* | *SlHAKAI* | Solyc09g013120 | 424 | 6.80 | 46.47 | nucleus | AT5G01160 |  |
| FIONA1 | *Oryza sativa* | *OsFIONA1* | LOC_Os02g02880 | 466 | 7.58 | 50.74 | nucleus | AT2G21070 |  |
|  | *Sorghum bicolor* | *SbFIONA1* | Sobic.004G018300 | 468 | 6.19 | 51.22 | nucleus | AT2G21070 |  |
|  | *Zea mays* | *ZmFIONA1* | Zm00001d053909 | 515 | 6.19 | 56.26 | nucleus | AT2G21070 |  |
|  | *Arabidopsis thaliana* | *AtFIONA1* | AT2G21070 | 513 | 8.50 | 57.53 | nucleus | AT2G21070 |  |
|  | *Phaseolus vulgaris* | *PvFIONA1* | Phvul.002G189600 | 444 | 8.39 | 49.37 | nucleus | AT2G21070 |  |
|  | *Solanum lycopersicum* | *SlFIONA1* | Solyc01g098460 | 424 | 6.80 | 46.47 | nucleus | AT2G21070 |  |
|  | | | | | | | | | |
| Name | Gene ID  (Phytozome) | Sequence | | | | | | |  |
| *OsMTA* | LOC_Os02g45110 | Amino acid sequence:  MEAQADAGGDDLAAMREQCRSLEEAIGFRRETQMGLVASLQRLVPDLVPSLDRSLRIIAAFNDRPFVPTPNPDGGHGKSPAALKPHHRRALPDPARSTRRKTSPGSSPASVAAAPGGLDAVRTMVAVCLLELVPFAEIDAAALARRLQAESSSASEAERTALADLAAELGGSAASAVVLALRRIAEDTGGVQIEEAMIGGKSMTMVWAIDRNKLLKELPESATLPLLQPPPAPQMPPSETDAGSAMIPRTPQQQQPQPDMWPHSMPPIFPRPRGMTMQGMQRVPGVPPGLMPLQRPFMGPAGVITMGGGVGPSPNQQKQKSEEDELKDLELLLNKKTYREKQNTKTGEELLDLIHRPTAKETAVAAKFKTKGGSQLKEYCTNLTKEDCRRQSGSFVACDKVHFRRIIAPHTDTNLGDCSFLDTCRHTKTCKYVHYELDQTPDIPPMMAGALAPPRQIRLQRAEYCSEVELGEAQWINCDIRNFRMDILGQFGVIMADPPWDIHMELPYGTMADDEMRTLNVPALQTDGLIFLWVTGRAMELGRECLELWGYKRVEEIIWVKTNQLQRIIRTGRTGHWLNHSKEHCLVGIKGNPLVNRNIDTDVIVAEVRETSRKPDEMYPMLERISPRTRKLELFARMHNAHAGWLSLGNQLNGVRLVDEGLRARYKAAYPDSEVQPPSPPRASAPIDGDQGTSQKPTVSDGERPA*  *Cis*-element sequence (2000 bp before start codon):  AAACTTTTCCTGAGATCACATTGAGTAGTAATTCAACCTGATAAACAAATTGCACTCAGCTTCGTGGGAGAGGCAGGAACTCCACATTGAGTAGTAGTTCAACCTGGCAAATAAATTGCACTCAGCTTCGTGGGAAGGATATGCATCCGATACCGAGATCTAACGATTAGTTGAAGAATCGGCGTGTCTGCCAAGATTGAAGTGCGTGATGTATATGAACCGACCCATATCAAGATCTAACGATTGGTCGGTTAGGGATGGAGTTACCGTCACCACTATGTTTTATTTTTTAAAGTGTATATAGTATTAGTATAAATATGATTCTCAACGAAAAGATTAGCATTTTTTTGTCCATTAATTTGCAGGACACCAAATATGAACAGTTACAAACTATTAATTGAGATCAGAGTTGCTAGCTAGATACTACGAATAAAAACTTCTCCAGGCGCACACCATGTTGAGTAGTAATTCAGCCTGAAATTTTACTCAGCGTAAATAATCCAATCTGGCGGATAGCTTCGGCGCGAATCTCTGAAGCATCGGCTTACCCGCGACAGCTGCGCCGGACAGGACTCCGCCGAGGATGCCGGCGACGATCATCGACCTTGGGCCGGACGCGATGCGGTAGAGCGCCCCGGCGCTGGCCCCCGCGGCGACGGTGTTGACCCAGTCGTCGGCGCCGTCGCGCTGGTCGCGGACGAAGCTCTCGGTCCCGGCGAAGAGCATCGCGACGATGCCGAACCGGTTGCCGAAGGCGCGGCCGACGGAGCCGCTCTGGTTGAGGGCGCGGTTGATGCGCAGCTTGGCGGACTCCCCGCGCTCGGCCTCGGCGGCGGCGCGGCGGAGGCCCACGAGCGCGCCGGACGTGGCGCCGGCGAGGTAGCTGACGCCGGTGTAGAACGTGAGGTTCTCGCCCCAGGTGCGGGTCTGCCGGAACTTGTCCTCCTCGAAGAGGCACTCCGGCGAGGTGGGCAGGTCGTAGAGCGCGCGGTACGAGGTGGGGAAGGCGAGGCCCTCGGGGCGATCCGGCGAGTAGAAGCGGCGCCCTTCCGTGGGCTCCTGCTGGGCGTCAACGGCGCGGGACATGGCTGGCTGATCTAGGGTTGCCGTGGGCTGCTGCTGCCGCTGCGTGCCTTCTGGGTTTCTGGACGATCGGTTTTGGTTGCCGCCGCCGTGTGCCGGGGGTTTAAGAGAGGGGAGTGGTCTCCGATCTGCAGTCTGCGCCTTCGCCGTGTCGCCGAAGAAAAGGGGGAGGGCGGAGCCGTGCGAGATGCGGACTCGAAACCCAGTAGGAAAAGGAACCACAACGCGACGCCACGGTTCCGACTTCCGATTGAGAAAAGAGGGTGTGGGCCGGTGAGGTGGGCCGTGGGTGTCGGCCTTCAAGTAAGTGGTCTGCCAGCGCGGCCTCCCTCCGCCTCCTCTCGGCCTCCACACAACTGTGCACGCAAGGGGCTAGGCGGAATAAGTCCAGTAGGACTCCCTCAAAATATAAGTCCAGTTTATTTGAATTCCTTAATCGCAAAGATATTTGAAACTGGCTCCGTCGTGGAATAAGAGAACATAGTATTTGCAAACGAAAAACAGTTTGTGAATAAAATTTTTATACACGTGTTTTTAGCGATCTAAAAGTAAAGACTGAAAAATAAACTTCGATGAAAAAAAACCCAAAAATCAACTCCAAATTGAGAGTTGAAAATTTAAATTTTGGCTGATAAGCATAAGCAAAAGAAAAAGATGAGGCTGTGGTGTAATTTGACTTCATAGATTATTGTAAGAGTGGTTTTCGCTGAGGTGGCAGGTTAACTTGTCTGCTTTCGGCCAGCTATTGGGCCCGCCACCGCGTCCCCTTTCTCAAATGGGCTCGACTTTATAACAGGCCGGGCCGTGCAAGCGCCACGTAGCTGCACAAACAAGGCCGTATCGCGTCGTGTCAAGCTCGATCTAGAGTTGCAAGACCCCCAAACCCCCGTGACCTCTCCGCCGCAGCCGCAACAGCA | | | | | | |  |
| *OsMTB1* | LOC_Os01g16180 | Amino acid sequence:  MFLQLADYHLRMDSRSPKIPRRSPDSKDKDSDRNKERDAKNDWDSSRAYGSETDCKEEMCDSNKRKGLTMGEIVVDNSRSVDSCHETELHVLRDDRQDKSVEIKDILHDGVAKSDYAQRQIDLDSERRNGTGDNSRVDVLRDDKLDSGRDRNWSDRTREPEGSKDYVRNRQWQDSKEANDSEWKNAHERLDGGSFHGRAGYRRDSRGRSESIRGSSTYGGRYDSSDSIEIRPNNNLDFGREGSVSGRRYDVGAHRDATPGTNGDKSANPEADQSGSTSTISQFPQHGPKGDRSSRGRGRPNSRDSQRVGGTLPIMPPPFGPLGLPPGPMQHIGPNIPHSPGPLLPGVFVPPFPGPLVWPGARGVDVNMLSVPPNLPIPPVAAEHRFAPSMGAGPGHNIHLNQIGSGIGAPTNVSGLSFHQLGTQSREMAHDKPPAGGGWTPHRNSGPTRKAPSRGEQNDYSQNFVDTGMRPQNFIRELDLTSVAEDYPKLRELIQRKDEIVANSASPPMYYKCDLRQHVLSPEFFGTKFDVILVDPPWEEYVHRAPGITDHIEYWNAEEIMNLKIEAIADTPSFVFLWVGDGVGLEQGRQCLKKWGFRRCEDVCWVKTNKKNATPSLRHDSHTILQHSKEHCLMGIKGTVRRSTDGHVIHANIDTDIIIAEEPTDGSTKKPEDMYRIIEHFALGKRRLELFGEDHNIRPGWLTLGKGLSYSNFNKEAYIKNFADKDGKVWQGGGGRNPPPEAPHLVVTTPEIEGLRPKSPPQKN*  *Cis*-element sequence (2000 bp before start codon):  GCAGGCGGACGGCCGGCGAGTCTTCCCTCACGCGCGGACGCCGGTGAGCTCCTCACATAGTAGGCGGACGGCCGGCGAGCTCCTTTCCCACGAGAGGACGCCGGCGAGCTCCTCCCCCTGCGCATGGATGGCCGGCAACCTCGCGGGACTGCCGGTGGGCTCCTCCCCCGCGCGTGGACGGCCGGCGAGCTCCTCCCCCGTACGTGCACCGTCGCCGTCGCCGCCGCCCTGCCTCTAGTGTTGGAAGGTGAGAGCAGTGTAAAAGATGAGAGAGAGTGGGGGAAGAGGTGAGGATGACATGTAGGGCCCACATGTCCGTGGGTCCCACTATTTCTTTTTTTTAATGACACGTCGGTCCCACAATTTTTTTATTACTTTTGTGCCAGATAAGCGACACGTCGACGACACGTGGGACGAAGACCGAGTCAACACCGTCATATAGGCGCCACGTCAACCAAAACCTCTTCCAAAACCACCCAGGGATGGGATATAGTTTGCATCGGTTTTAATAGTTGGGGGAGTCAATATACCTGGTTTTGTTTTGTGGTTGGAGGTTATGAATCATACTCGGACCATAGTTGAGGGAGTCCAACTATACTTTTTCCGATGATATACACGTGCTTGTTTCTAGTGGGACGTATCAGATAGCCCGCAATCATATTCCGACCCAATTGAAATCTGATACCGGGCTGGGCCGTATCCATTACTAGAATGAATATACGGCCCAATTGAAAGCTTACAGGCACGGCGTGAGCGCGGCGTGGCTTAGCCCAAATCGACCGACCGATCGATTCCAGGAGCGTCGAGCGCCTCCGCCGCGCTCCGCCGCGCTCCGCCGCCGCCGCTGCTCTCCCCGCCCCTCGCGGGGAAGTCGCGCGAGGCGTCGTCGGCGGAGAGCGCGCTGTGGTGCGCCGCCGCCGCCGCGGCAGCCTACGCCCTTGTCCGTGTTGAATAGTACTACTACCATATAAACAGTCCGTCGCCGGAGGTTCTGCTAGGGTTTCGGCTAGCGGGGTGTGAGGTGGGCCGTCCGCCGCCGCCTCGGCTTGGAGCTTGGCCACCCCCTCGGCAAGGCCGGCGCCGCGCTGCTCCGCCGCCTTCCCCTCTCCCAGCCAGCGCGCGCCGCCGCCTTCCCTGAGTTCCTCCCTGACGGATCCCGCAGGTTCCTTGCTATTAGTAATCGTTAATTTTTTTACTGGGTGATTTCATGTTCTTGTAAAGAATTTCAGGGTAGAAATCCGAGTAATTTCTTCTCTCTCTGTTGATGTTTTTTTTCCTTCAAATTTTCTGTTCATGCGTATCTACATGTTTGTACAGCCAAACTTGAAACTGTGCATGCGCATCACCTTCATGCCCAAAATTTACATGAATTCCTTCCAAACATAGTAAAGGAAATGGTAACCAAACAATGCATTCCTTCCAAACACAGTAAAGGAAATGATAACCAAACAATTTCTTCCTTTCATTACTGTTTGCGGTTTCAGACTCTTACCAAACACCACCTTAATTTAAATCTTGCTCCAGTTTTGCACCTTATTTGACATGTATGCACTGTTGTATATTACCCAGCCCCATTGTTAAAGAGGAACGTAGTAGATGTGTACTACTATACCACAGTATATTATCTTTTGGGGTTTGAAAGTAAGCCTTGTTTCCATGAAAAAAATGTTCCGACATCCTTCCTTTGTATCGAAGACCTTGTTTCAATTAGTTGTTTACATATTTAACGCAAGCTCACTTGTGTGGTACGAATTCTGTAATGCTATTATGTGCTGCAAGTATAATATATTTTACCATTCTTTTATGTAGTGAAACCTGAAGTTTTTAGCCAATGGTTTCTGAGATCAGCTGTATTATTCAGATTTGTGTAGTGAAACCTGAAGTTTAGCCAATGGTTTCTGAGATCAGTTGTATTATTCGGATTTATGCTGTGAAACCTGAAGTTTAGCCAAAGGTTTCTGAGATCAGTTGTATTCTTCGGAGGAGGGATAACAATATTT | | | | | | |  |
| *OsMTB2* | LOC_Os03g05420 | Amino acid sequence:  MDSRSPKIPRRSPDDSKDKDSDRNRGRDEKNDWDSSRTYGSETDCKEERCDTNKRKGSAMGEDVGDDSRSVDRSHETEVHVFNDKQDKAVEIKNILHDGVGQSDYGQRQLGLDNERRNGTVDKSRVDAHIDDKLGSGRDRNWTGKTQEPEGSVDYLRSCKSQDSKEASDSEWKNAQERQDGGGFHGRVGYRRDFRGRSESTRGSSTYGSRYDTSDSIEIRPNNSLDFGREGSVSGRYDVGVGAHRDVTYGTNGDKVTNSEPDQSGSASMISQFPQHGHKGDRPSRGGRGRPNGRDSQRVGVTLPIMPPPFGPLGLPPGPMQHIGPNIPHSPGHPLLPGVFVPPFPGGPLLWPGARGVDVNMLSVPPNLPIPPPVAGEHSFTPGMGAGPNIHLNQFGSGIGAPTNMSGLSFHQLGTQSREMVHGKPPVGGGWTPNRNSGPTRKAPSRGEQNDYSQNFVDTGMRPQNFIRELDLTSVAEDYPKLRELIQRKDEIVAKSASPPMYYKCDLRQHVLSPEFFGTKFDVILVDPPWEEYVHRAPGITDHIEYWNGEEIMNLKIEAIADTPSFVFLWVGDGVGLEQGRQCLKKWGFRRCEDVCWVKTNKKSATPSLRHDSHTILQHSKEHCLMGIKGTVRRSTDGHVIHANIDTDIIIADEPTDGSTKKPEDMYRIIEHFALGKRRLELFGEDHNIRPGWLTLGKGLSYSNFNKEAYVKNFADKDGKVWQGGRNPPPEAPHLVVTTPEIEGLRPKSPPHK*  *Cis*-element sequence (2000 bp before start codon):  AGCACAAATAAGAAGACAGAAGAAATATTTGAATATGAACTGTAGAGTCATCCAACTGTCCATGCCTAGTTGAACCTGCACAAATTACCATTCTCCATACCTCTCAAGGAGCTACTGGCCGCTGTAAACGTGGGATGCATCGAATCGCCCGTCACTGATACTACGGTTGCATATCGATCGGAAATGGTGAAACCGACTAAATACCATGTCAAAGTCCTCTTGGCGGCTAACCAAAGATGGTTTCGGGTTCCCATATAAAATTACCCACCCACTCAGACAACGGTACAGGTAGCAACAAGCGCGCGAAGCATCAGAAGGGTGAAATCACTGGAGAAATGCGATGATTTAGCATTAGAGAGATGCCTCAGCTAACCTGATGATTTTCAAGCATAACTAAATGGATGGGACATGACTGATTTCTCACAGCCGAGGGAAAGCTTACCCCGAATCCCCCCAAAATTTTTTGGTCCGGATGGATGTGGATCTCCCGTAGAGGGATCCACCAGGCTAAGCGTCCGAGAAACAGCCGTAGGGTGAAGAACGGCGGAGCAACGGCAGGCGTGTGCTTCCGTCGTGGTGGTGACCGGAGAGAAGCGGCGGCGGCGGCCGCGGCGGCGGAGGCGGCGTTTGGGTTGGGAGGAGGAGGAGAGCTCGTTTGGTTTTTAGTCGGAAAAGAGAAGGCGTTGATCGTTTTCGGTGGGCCAGATGGGCCGAATGTATATGTGGGCCAGATGGGCCGAATGGCCAACCCCGACCCAAAATTCTCGACGCGGTCCCGCACCAAAACCCCTGCGTCGCGTCGCGTCGGAGTCAGCCAACCACCGGCGGCGGCGGCGACTACCTCGCGCACGGCGGACGGCGGCGAGCGGCGATCTGCCTCCGCCGCGCTCGTGCCTCGTCCCGCCGTGTAACCAAGGCTCGTCTACTTGAAGCGAACCCTCACCTCCCATCCTGGTAAGCCCTAGTACTCCTTTCTCCCCGCCTTCAGTTCCCACGCTCGAAGTCTCGATTGAACAAAGGCTTCGCGCGGAGAGTCTGTCCTAGGCTTTTGCTGTTGGATCGGAGTGTTTAGTTCCTTTCGATTCTGCTGCGGTTGAGTTCAAGCGCGATGCTATCATGGATTTTTGCTAATGATTTTAGGGGTTTAGGGTTGGTGAGCGGTGAAATCGCGCTCCGTGTTGAGGAATTTTGGTTTCCTCACTCCGTGTTGAGGCTAGAATTTCGTATTTTGATCACCGGAGATGGCTAGGGATCCGTATTTCTAGCGTTTAGGTGCAACCTAACGATCATATGGCCGCGGTGGGTAATAGATAATATAATGCCGTTGCTCTAGGTTTCCCATGCCCTCATTATGTCGTGTTTTGTGTGTATTTGCTCTAGGAGTTTTGTTCGATTTGAGGCAGTGGAAAAATTATAGGCTTTTGGGAAGCTTCTGCTTAACTTGACCTTTGCAATTACTAGTAGCGCATGATCTGGGGGTAGATATTGTCTTCTTAACTTATCTTTTGATTATATTCTGTACAATCAGTTAATTACTTGTTAATCACTTATACAATGTGTTGTGTTAGCCAATCTATTATACAATGTGCAGCCTTTTCCAATTTATTTTAGGTCTTGCTCCAGTTTTGCACCTTATTTGACCTATGCAGTGTTGTATATAACCCAACCCGTTGTACTATGAGGAGCATAGTAGATGTGTTTATCACAGTATGACTATATTTTGGTGTTTGAAATGCCTTCTTTTCCTGAAAAATAATGTTTGGATATCCTTCCTTTGCATGAAAAGCATTGTTCATTTAATTCTTCTCTATCACAAGCTCACTTGTCTGGTTCAAATATACTGTAATGCTATTTATATGCTGTAAATATGATACGTTTTACCATTTTTTTTATGTAGTGAAACCTGAAGTTTCTAGCCAAGGTTTTTGAGCTCAGTAGTATTATTGGGAGGAAGGGTACACTATTTTGCATTCTTGAAGTTCACTGACCATCATTTGAGA | | | | | | |  |
| *OsMTB3* | LOC_Os10g31030 | Amino acid sequence:  MEGSDSSRSRDKRDADDDVDARSSWKEDDEHEDVEDRKNRSGKSTRYIYDDEGDEDDYDVRRESRVSKVPRRSPEERSERRLSDGYKDRDGDSSRRRREDNNDWDSSRRSGSRTSGHDVSRSKSRSSDRTSSDRADTRDSRSSADQSKNRSAREAHDYRNELSERWEDTERRKGSARTDKNDQDRRSIDPRYDSPPARDDRIVDSVDNTRQNTLHYNAKSEESDEKCMDQVEGTNRISDNVETKDMLPYVDKDGHALSRDGRNKEARHHREKDDGDQGHSDSDNERNISMKEKIRVDAHGDYKSYRGRDRNRELEGSKEHWGSRQRHDSKEPNDYDVGAEWRHGQERLDGGNFHGRSGYRKDSRGRYESSKGPSSYGNRYDSSDSIEIRPNRNLDFGRESSVSGRRINMGSLQDLTPGTSDPSEENKRNYGNGEDAQERYYDDVQNMDGKIPSDSHTGRGGAIASNNSGAGPSGSGSIISPTPQQGPKGSRPSRGLRGRPNVRDPQRMGLPVPLMPPPPFGPLGLPPGPMQPIGPNMSHSPGPLGPGVFIPPFPGHLVWPGARGIDVNMLSVPTNLPIPPVAGPSFTSSVAAGPNHSIHLNQTGSGLGSPANAPGTGFNPLSTPNHEILQDKPPAGWTPQRGPAGKAPSRGEQNDYSQNFVDTGMRPQNFIRELELTSVVEDYPKLRELIQRKDDIVSNSASAPMYYKCDLKDHMLSPEFFGTKFDVILIDPPWEEYVHRAPGITDHIEYWTPDEIMNLKIEAIADTPSFIFLWVGDGVGLEQGRQCLKKWGFRRCEDICWIKTNKKNATPGLRHDSNTLFQHSKEHCLMGIKGTVRRSTDGHIIHANIDTDIIIAEEPTDGSTKKPEDMYRIIEHFALGRRRLELFGEDHNIRPGWLTLGKGLSSSNFHKEAYIKNFMDRDGKIWQGGGGRNPPPDAPHLVVTTPEIESLRPKSPPQKSQQQQSMPPMGSSSSTNRRSVMNSSQIVVTVVGSETMMPSPWSSNPMSGFGMPE*  *Cis*-element sequence (2000 bp before start codon):  GATAACGGAGCAGCAGCCGGTGTCCATCCTCGGGAACCTCGCGCAGCAGAACATCCACGTCGGCCACGACCTCGACGCCGGCACGGTCACCTTCGCCGCCGCCGACTGCGCGGGGAGCGGCCGGTCGGCGGAGCAGACGAGCGGCGGGCAGGCGCGCCGCAGGAGAGGAAGATGGCGCCGACGGGGATCGGGACGGGGAGAGCGAGGACGTCGCCGCCGTCGTCGTCCATGGCCGCAGATCTTTCGTCCGCGCCGTCAGCGGCGTCGCTGGCGGGTGTGTGTGAGCGCGGGGTAGCGGCGGGTGGAGCAGGCGGTGGTGGGCGGAGCGGGCACGACGCCGAAGACAGCTTCTTCCCGCGCCGGCGCCTCCGGCCGCTTCTCCCTTCTCAAGCGTTGCAGCCGCCGCCACCTCCCTTCTCCCGTGTCGTCGTCGCCGCCGCCGCCGCCTCCCGGGCCTCTCCGACGGTCCAACCCCGAGGTCGCACGCGCCGCCGCCGCCGCCGCCGGACCTCCTCCCCACCCGCCAGCCGCCCTACAGAGAGGAGAGAAGTGAGAGAGAGATGAGGAAGGGAGAGAAGAGGGGGACAGATGACCTGGTATTGTGACATGTGGGGCCCACGCTGACTCAGCCGCCACGTCAGATAAAACCGGGGTCAAAAGCACCGAAGGATGTAAAGTGAACGGTTTTGTTAGTTGAGGAACGCCCGGTATCTGGTTTTGCGGTTGGGGGATGATTTTGTAACTCGATGATAAGTTGAGGGACCTTGGGTGTACTTTTTCCTACTGCCCAAAGCACCCGCACTGTTGGATCAGCCCACGAGCCCGCACTTCCTCCACGGCCTAAATAGCAATGGGCCGACGATTGCCCAAATGTTAACCGGCCCATGGACCGGCCCTTCTAGATCCGTCCTCAACTACGGGGGACCTGCCTGTGCTTGGGCGTGTCCGTACGGTCGCCGCCCGCGGCCGCGCTGCCTCCGCCTCCGCCCCCCGCGCCGCCGCCCGCACGCTCGGCGCGCCGCCGCCGCCCAAACCCTAGCTCCATCTCCCGCTTCCCCCAGCCGCCGCTCTGCTCCCCGCGAGCTCCTCGCCGGACCAAGCCGCCGCGGGCGCCACTCCCCCCCACCGCTAGCCTCTCTCTCTCTCTCTCGTCGTCCGCTCGCCCCGTCGCCGCGCGTCGGTCGCAGTCGCGGTTGCTCCGCAGGTATAGGTTCACATGCTCTTACCTCCAATCACATGTCGCGGTGGATCGCTGGTTCCATGCTCTGAATCACTTAAGCTCAGTGTGCTAATTTTCCCACGGTTGTAGTTGGAGTAAGTTGAGTAAAGAACAAGTACTGGTCGGTGTGATATTTAACTGAAGTAAATCAAGCAATGAAAAGGTTCACCTAACTGTTTAGTCTACCTAGCTTTATTTAGTATGAATCCGGGTATTCTATCCCTCTAAATATGGAAACGGATTCACATAACTCCTAGGTGTGGTTCTGTAGGTGATTCGCCGCCATGGCGTCCGACGTGGCTAGCGGGCGTTGATGATTTACGTGATATAACTGCAAAACCGGTAGCTTGGTTGTCATTATAAATTTGGTATCAATATAATTAGAAGTTTCATGTGGCTATAGAGATGGATTTGATTTTGATTCGTATTTATTACCTCTTGAGATGCACTCATAGTGTAGTGGCAGGGTTGGCTGGCATGCACCCCATCCGCCTGGGTTCGATTCTCCTCGAGCATGCTCACCGGAAATATTTTGCTTCTTTCTTCTTAAAATCATTGACACCAAGCCCTTCCTTGGTGTCCAACTTTTTTATTTATTAGCTCTTCTCTTTTTTGGTGATGATAGCATATGTCTTCAAAGTTGCGGTTAGCGTTTCCGGTCAATAGGGTGTCCAGTTGTGCTGCGTATGAATCCCATATATGGCGTTGTTTCTGCAGGAAATTATAAACTTTCCTGTTGGCTTAACTCTGTTGAGCATATATGCCTTAGAGAGCTTCTGCA | | | | | | |  |
| *OsMTC* | LOC_Os03g10224 | Amino acid sequence:  MGISESDELRAFEATGIYRLAESGAAFLDPVRILNASYRRFRLVPSAYYSRSFGTSRQGGEAETERTGEASPERKKRKRKRQRQPKPRELNEVERMAEARHQEARPLLSSAHKSLLKAKDLLEFLPRMIKEDVRMLDVESNLEKNLVELGSSWRAPLCEMTLCFQKSSGEDSEEGICHKTSTPLFNSTISIEENDDAEGEFQDRRYILPRRCCFLMTDLKHVRGLIPDNYNQGYNLIVVDPPWENGCVRQKVAYPTLPNRHFLYLPVQELAHPAGALLVLWITNREKLWKFVEEELFPAWGVKDHTVFYWLKVKPDGSLIGNLDLLHHRPYECLLVGYINLNKEAVRGSKFKFLEERRVIMSVPGAHSRKPPLQKLLSEYIPGPKPARCIELFARELVSGWTSWGNEPLRFQDAMYFFNKQEHDVHS*  *Cis*-element sequence (2000 bp before start codon):  ATAAAAATTTGATGTTTAGGAGAATATTTATGCAAACTTTTTACACTTTAACTATCAAATTTATATTATAATAAATTTATAAAATCTAATAAGCTTATGACAGTACTTTTCAAGGCAAATCTACACATATTTCTTTTTTACTTTTAAAAGTTTGACCAAATCTTGTCCTAAACATCAAACGTTTATGCCCGGAGGGAGTGCCAGTTAAATAATAAACTTTAAACATAGCATTGCAAAATCACCTGCAGCAGCCATGCCTGAAGCAACACCTAGTGCTTCTAGTAAGCCAATCAACATGAAGCGTGATTTTGGAAGTGCTAGCATTTCTTTAGTGACAATGCCAGCATGATATCTTATAAAAAGAATAGAGAAATATACTATAACATACCTGCAAAAACAGATAAGCATATGAGCATCTGCACAACTATTGTGAAGAAATCATCTATCTATTACTTCCTCCGTTTCATATTACAAGTCGTTTTAACTTTTTTCCTAGTCAAACTTTTGTAAGTTTCACCAAATTTATAGAAAAATTCAGTAACATCTAAAACACCAAATTAGTTTCATTAAATCTAACATGGAATATATTTTGATAATATATTTCTTTTGTGTTGAAAATACTACTATGTTTTCTATAAATTTGGTCAAATTTTAAAAAGTTTGATTAGGAAAAAAATCAAAACGGCTTATAATATGAAATGGAGGGAGTATATTAGCATAGTAGTAAAAAAAACTAAACCTCCATGTATTCTCTTAATGGTTCTGAAGTACTTCTAGAACGTTAGGCAAGTTAAAAAATATATACTTCATCCGTTTCACAATGTAAGTCATTCTAGCATTTCCCACATTCATATTGATGTTAATGAATCTAGATAGATATATATGTCTAGATTCATTAATATCAATATGAATATGAGAAATGCTAGAATGACTTACATTGTGAAACGGAGGGAGTATATCTTACACCAGTAATCAAGGTGGGTTCAGACCGTAGTGATATAGACTTGATCAAATATATCTCAATCTTAGAACTTACAATGACATCCTAAGAATTAAAATTGTGCCTTTTGAACTAAGCGAAAGAGCTGTACAATAAACGCAACTCTATCTCCATATAAAAGCATGTATAAGCATCCTGTAGTTGTATTGTACGGAAAAGCAAACGAGATGGTGTGCATAACACACTTACATTAGTTGACATGTAATTCTGAATAAGTGTTGCACCTCAGAGGTGGAAGCAAACAGACAAACTTAAAGTTATCATTCCAAATAAAACAAACTTAAGAGTCAAAGCGTTAACACGCATTTCGATTGAGCTCAAAGAATGTACTTGTAGAAGACTAGACTGTAGTACAACAATACTCCACGGCACAGATGACACAAGCGATCACAAAATCCTAACAGTGAGCCGTGGTATATTGTAATCTGTGCGAAGCATCAAAATTACCCAAATGTGGTGGCTTGGGCGAGGAAGAACGGGTAATTCCTCATGGGCACGAGCGCCAGCTTGTACAGCACCCGGTTCATCACCGCGAGCACCACCGTGGCCGCCGCCGCCGCGGCGATCCCCGTCCCACCCCCACCCTCAACCTCACCATCGCCCCCTTCCCCCGAAGCCCCAGCCCTCGCGGCCGCCCGCGCGCGCGTCGTCGTCGTCTCGACGCGCCACCTCCCGGCACCATCCCAACTGGCGGCCGCGACCGCGAGGCCCACACGCCGGGGAGACGGGAGGGAGGCTCGGGGGGAGAGGGACGCCGCGCTCCATCGAGTCACGCGGCGGAGGGAAGGCCGCGGCGGCGAGGAGAGGAGCAGCGCCATGGGCATGGCTTCGGTGGGTTTGAAGATTTCGCGGCGGGTCAGTCGGTTTTGCTTCGGCTTCTGTGGACGGAGGCCACTGGTCACTGACCGGTGGGGCCCACAGGATTAACCCAACCCACCTGGCCTTTCCGTGTCCGTAGCCCACGGCCCGAAACCCCGACTCCCCGCCGCCGCCGCCGGTG | | | | | | |  |
| *OsFIP37* | LOC_Os06g27970 | Amino acid sequence:  MADSPSPRLDEEDAFGRDFNSSPSPTAPPARSGEKRPFGDLDDDDEDVFASKKGKTKVEESAPGAATGMILSLRESLQNCKDNLASCQVEREAAKSEVQKWHSAFQNIPAVPAGTNPDPVSVVSYLNNLKSSEESLKEQLEKAKKREAAFIVTFAKREQEIAELKSAVRDLKTQLRPPSMQTRRLLLDPAIHEEFTRLKNLVEEKEKKIKELQDNVAAVNFTPSSKHGKMLMAKCRTLQEENEEIGAMASEGKIHELGMKIAVLKTRNNELRNQFNELYKHMDGLTNDVERSNEMVAILQDELETKDVELRRLKEMLAQKEATDENKIPQENDVAGDDIVAAAESQPIKVET*  *Cis*-element sequence (2000 bp before start codon):  CAACCTCTGTTGCCTTCCTTGTAGTGCTCTATGACGGGTGGTGGATAGGGCTAGTGAGCAGGAGCTGGAGGCCGACCGTTGTTGTTCTCGAGCCTCTCTCACACCATGGACGACTACACAGCTAGTGGCAACTGGTTTTTTAAACCAGCTACCACTCCATTTATGAGAGGTCCTATCCACCTATAATTCAAATAAAATTTTCTCTTAAACTACTCATCCGATCTACGATCCAATTACACCGTTATGTTCGTAACAATTAAATCGTTATAACAAGATCTCACATGATTATATTTTGATGAAAAATTGTAAATTACTTTTATAATATATCTAAATTACTTTTAGATTTCACTAAATTACTTCTTAGACATATAAAAGTAAATTCAATAAAGTCTAAAAGTAATTTACATATATTATAGAAGTAACTTATGATAAAAAGAAAGTAACTTTAATGCATGACTTTTTTCATTGGACGTGACTATACAGCTAGTGGTAGTTGGTTTGTCTAAACCACCTACCACTCCATCTAGTGTCTCCTTTCAACTTGAAAAATGCAATAGTATGTGTCTTTTCTTAATCTCTTTAATAACTTATATCTTTTAAATCACATATTGTTTTTAAGATCTATTTGCATCATTATGCTCCACTCAAATATGTGACAAAACGAGATCCATATTGATTATATTCAAACAGTTTATTAATTTAAAAGTACTTTATTATATGTTAGAAAATAACTTCCACGTTATCAAGAAGTAACTATTACATCTTATACATGATTAATTCTCATTAGTGATTCCATCAATGTTATTTATAATTCACCAAAGGTTTTATCTTCTTTTCTCATTCTACATGAAAAATAAAAATAAAAGGAAAAAACAATCGGTGATTAAACAGATTTATAGAGAATTACTTATGAACCATGCAATAGTTACTTCCAATTAACATTGAAGTTACTTTTAAAAATCGTTAAACTTACATGTGCTGATCTGTGCTGATTAAATTTAATTTGTAGATAAAGTCATATTTTTATCCCTACAACGAATTTACTTCTAATGTCGTGACAAAGTTACTTCCGTTTTATTTTAAGTTACATTTATAAGAGATAATATATGTAGATTACTTTTAGAGTTTATTGAATTTACTTTTATATGTCTAAGAAGTAATTTAGTCAAATCTAAAATTTATGTATATGAAGTAACTTACATATGTAATAAAAGTAATTTACAAATATAAATATTTTTTTCATTGTAATATAATTATGTAAGATCTTGTTCTAAAGATTTAATTGCAGCGAACGCAATGGTGTAATTGAATTATAGATCGGATAAGTAATTTAAGAGAAAACTTCATAAGAACATAAAAAACCTAATAGCAAAAAATACTTGCATGTATGTGAGGTGCAGTAGTATTTCTCGCATGTATTGTCGGTGTCGCTGTTAAGTCTAACTCAAGATGCCTCTCAAATAGATTTTGCGTTCACACCACCCCCTCTAACCCGCCGCAGTGGTGGGGAGAAGTTAGTCGCCATGCGATCATTAGATATGCCATGACCAGAGAAAAAGGGAGGGAAATGAGGACGATATTTGATTCAACTGTGCTATTTACATTTTCTTCAATTTTGATTGCAAATGCCAAGTAAGCGTCACATAAACAACATGTCAACGCACCTCACGTGTAATGAAACTGCGTTTGGATTTGATAGGTAGCTCTGCACTTGGTCTTTCGGTTGAGAACACGACTGAGACTCGACTAATAGTCAAGGGAGGTTAACCAGACTTTTTCCATAAAAGAAAATGGGAGAGTCCTTTTGGATTAGTTTTCGTTGCCCTGGTGGCCAAAGCCCAAAAGTGCAGACGCTGAGACGCGGCCGTTCCCCTTCCCCTTCCTCGAGACCCAAAACCTAAACCCTCGCCTCGCCGTCGGTGTGCCAGCACCCTCCCGGCGGCCGGAGCGGCGCGACTTCCCATCTAACATATTGCTTCCGGCAGCCGCCGCCGGACACC | | | | | | |  |
| *OsVIR* | LOC_Os03g35340 | Amino acid sequence:  MGRPEPVVLFAQTILHSQLDEYVDEVLFSEPVVITACEFLEQNASPSTPNISLVGATSPPSFALEVFVHCDGESRFRRLCQPFLYSHSSSNVLEVEFNGPYSSSEATAIAAFSMFVLFYQAIVTNHLVLRGTYRSLTLVIYGNTAEDLGQFNIELDLDHSLANVVSSPSEGKLEDLPPALHSSKFTFEESLSSLKPLSLQATELDLSIEVKKILLLALTMYQIPNVENLIPNLQSAVISAVLKYMPASTNCMSRNWNRDPANCFAEDNVDSQGTSNTLLMEASNELFDIWKNVNSIVDNITFDDNGLAFRLEELPTTKHLFTLFDSCFPYYRNCSLLDLECPFQPLVDLQSAVVNIVSNLPSEELSSDGVNFLSSASIELAELLKMINMCVPIEDPSPVLTARRICKFGHLEGLLSYNLTIGLITSSKYSFLQFDADPYMLSLIQEDASGNLERRELVGPTGHTLPMRAPATHAYSSTSAPTTLLCPCLPESSSGCGILPAPPSFNPCRCDKPSSDVAHGIPAIPHYCLLSADGLSFLLGQPEATELILLSLQDGEDMSKTECMTLRQAFVLLSKGFFCRPQEVAMITELHLKVEHSLFAYNLLNLKVSTLLSHIDDLNYLAVSVLLSSLSSYNDLDSVTNKNGGSPLGHAIFHSTAEILEVLVADSTASSLKSWIGFAIDLHKALHSSSPGSNRKDAPTRLLEWIDAGVVYKRNGAVGLLRYSAILASGGDAHLSSGNVLVSDSMDVENVVADPNNTDGQVIDNLLGKLVADKYFDGVALCSTSVVQLTTAFRILAFISEEKAVASSLFEEGAINVIYVVLMNCKSMLERLSNSYDYLVDEGAELSSTTELLLDRTHEQTLVDLMIPSLVLLINLLHILNETKEQYRNKKLLTALLQLHREVSPRLAACAADLSFMFPSFAVSFGVVCHLVTSAIACWPLYNWAPGLFHCLLENVEATNAAVPLGPKDACSLLCLLGDLFPDEGIWLWKVEVPSLTAIRSLSTGTVLGCQVEKHMNWYLHPEHVSILLVRLMPQLDRLACVIDNFATSALTVIQDMLRIFIVRIASEKIECAVVLLRPIFIWLNNKVDETSLSEREIFKVHQLLQFIAKLSEHPNGKALLCKMGVARILRKLLQECSSMCYMEDNMISDKGVYSNDLLMLRWKIPLLRSIASIFSTRPSSKEPTTVEELWNENACVEECSSIMYHLLMLCQVLPVGRDMFACSLAFKEVASSYSCRGAVTSIFSQIQTSNKDESQKSESETCHDTSKVDNWCGFFPLLKCWKRLLQYICANRPTDYLVEIVYALTLGAIALSQSGQNLEGTIILRRLFGHPSVPSSSEASDEVTFLLKTFQEKICQGFDNWSPYVGKPLLHQVRSSVRLLCSIIENSGPFTDSVRMVLEESTIPVGVFHNIVMTSHLMPSIDFVSVNDDPALLFTNAWKAFGDFAEPFGCQVSDFSKRMVWELPDCSIDKQLIPSQSARRKLALGDSASRRVRDNQTHEPSGQFSRGLNTPSASIGHTRRDTFRQRKPNTSRPPSMHVDDYVARERNIEGASSASNIVSSTPRGALSGRPPSIHVDEFMARQRERQNPVLAPSGDATQVRSKATLDDNVSTKPEKPRQPKADLDDDQEINIIFDEESGSDDKLPFPQPDDSLQSPPVIIGENSPGPVVDETENQQNGINLFSGTVVSESDEACETVISSQTAIRQESNIPSERKFSVSSPEKVMFPDHADESPFISPTTGLKVIPGYSTHAAQATLRQLPPNMHRKRSPHKLAESSVSSGSHGHDRTLYNSQPPLPPMPPPVSSTSLQNPDSIQRQPSSYIARDGPPPFPPSYLMQSFDACMPSFVGHQVQTENVLPSTGDSSSNALPSVDAKFLWSTLPVNRIPMEHLSSGSSTRPVSPLPLRPVLATQHAAMDSGPPGSLYNQGGSGVLQPSPPASLINDATLGTNPASGGALASNSLPSLASQYIIGRPSTPPFFGTPLQIQLSSGLAQSVSNPQPSLSSMQPRAPPPPPPQPHPSQTFQGSLQQPQEQPMPYPLNTIQPQVPLQFPNQLHVPQLQFYHQTQESVLQPIGQSAQQQMDSGMNLNHFFSSPEAIQSLLSDRDKLCKLLEQNPKLMQMLQDRIGQL*  *Cis*-element sequence (2000 bp before start codon):  TCAACACCTATCGGCAATTATAAAGCAAGTTAGTTCTTACAACTTTGACTATAATTTAGTAGATCAAACCTATAATTTAGTATATCAAACCTAACTGATGCAACATCAAGTAATAATAAAAATTGATAGGTCTAGACAAGCAAACAATTGATATTCATGGTGGTAGATACATTAACTAATCTAAAGCAACACTATAGTTTAGTCAAATATCACCATAGCCAAATTTGGATCGATCGATAGAAGTTAAATAATATTGTCAACTTAGATTAGCAAATGTATATGATAAATAATGCTTATATCTACTGGATTAGAGTTTGAGATCCATCCGATGGAAGCACTAGCCCCGACGATACAAACAAAATTGACGGAACTTACCTCGTTGCTAGACATCAAACTCAAACGATGCAGCCCAACTCGAAATAAGAACTCGTCAAGATAAAAACTAGGTGGCAATGTGCCAAAAAATATATTATTGAACTGTTGTTATTACAAAAGACCCTGGGTATATATATTTATACCCATGAGCAAATACTAATACTTATCGAACACAACGCAAACTTTTCTAAAGATAAAAGTAAAAAATTACAAGCCCAATACTGGTATCTAAAACACACTTTCCTAAAATATAGAAGGAAGAAACAACTAAGTCTGCTAATTGATAGATAAATCACCATGTCAGATTTCTCCGAACTTATTCTCTTCTGGATGATATTTCCTTAATTGACCAATTTTCTTATCCTGACAAGAACTCGACTTCCAGCTACTGACAACTTCCATCGGCAACTTTTATAACTTCCAAAGCCGATATCAATCGCAGCCGATTCCACTTGCTTGGGCCTTTAGCCGATGTAATCTATATTTCTTGGCAACCGAAATCATGCCATTATTTCCGTTTTTACTTTCTCCTTGACTGACAGTATATGCCAAATTTTGCCGAACGTCAGCACGTCCTCCTCCTCACCATCCTTCGTGCTCATCAGCGTGTCGATCTTGTTGGTGTCCTCCTTCTGCCAAATCAAGCACCCGTCGTCATCGTCCTCGATGCCATAGTAGGCGATCTTGCTAGAGTAGTCCTGGTCGGAAGCCATAGAAGTGGTGGCTAGTAGCAGCAGCTTCCTTGGTGACCCCGCTGTTCTACCTCCAAGCCACCATCCATCATTGTCCCATGTTTCTGCTGCATCTTGTCGGCGTGCGGCCGGCAGCACCATGCTTCATGCTAGGCGGGTACCTCATGGTACATGATGATTGGGAAGAGAGAAGAGAGAGGAGAGGGGGAGAGAGAGGAGAGGAAGAAAATGCTAACCACTAACATGTGGGCCCGACGCGACTCAGCTTACCACGTCAGCTAAAACCAGACATAATATTGGCTAGATGATCTTGTTTTTTAAATTAGAGGACCTATTGTATCTCGTATTAGAGTCCGCGACAACTTGAGGGACATAAATGAACTTATTCAGCGGCCAATGCCATATCCTAGCGAGAGCGTAATGGGCCTATCGACTCCTTGCGATTGTGGCCTTCTTTATCGATCTTGCTGCCGTCCCTGGCCTGCCTGGCTGCCTCGACACCCGTCCCGTCTCCTTCGCCCGCCGCGGCGCCGCCTGGGGCTTGGGGCCAATCACCGCGACGCGAGCGGCGGTGCAGGCTGCTTCCGCCGCATACTCCTTGCGGAAGTCAATCTCTCCGTCCCCTCGGCCTCGACTCCCGGAGGTCTCCTTGCGAGCTCGCCGACGCCCGCTGCCGCCCGTGCCGATCTCCTTCCCGCGAGCAGCGGTGCCGTCGTCCTGCTGCTTCCGCCTCCGGTGAGCACCCGTGCCGACGCATATCCTCTTTCTTTCCCATCCCCTCTCTCTACACACTTTTTCCCTCTTTGCTTTAACCTTGTCTTCTCTTCTAGATCTGGAAGCTCTTCTTTGCCTTAAACCCTAGTGCTAATAACAGAGTTTTTTCCCAAGAAAATTTGGGCACGTTAGGTTCCGCGTCCGCCCCTGACGAAAGCA | | | | | | |  |
| *OsHAKAI* | LOC_Os10g35190 | Amino acid sequence:  MLQIRLSKIGSSDSGAAASGAAAGAGGGVGGGGVGGAGAGAGGGPPKSASAAAGGAPESVTVACPDHLVIADLAVAKSLGAVTTSAVAAARTIGRRSRRPLGERVHICCRCEFPIALYGRLIPCEHAFCLACARSDSSCYLCDERIQKIQTVKMMEGIFICAAPMCLKSFLKRSEFDSHIPEVHANLLHNTPEREERNEPDAPNISRASGGDQRQSQMPEMSTARAPPRTGVSPSSSSHVQDRDDRSRYHHSRDQTPQRPPMLSRPPSFHGRHSYPPGDTPSENNPPQGFDRPYNWAHENAPGATPVRQESEHGSQDKQQMMPNAPFMFPPMPHQPNFMMPMNMNQPLMSNTSFNYPLQQDGNPQFFSAPFQMQLPDVGLDQGSASGVQPTPPGPLSFPEGLQRPWGMGLMGNPFQSMPLGQGMPEGAGEPQGGGGMVFLQGGFGVMPDGSMNSGIPGRDLSGQGDRGVLAQMPMPMQMQMSLPPPPPTQPPSAGQQTFSRT*  *Cis*-element sequence (2000 bp before start codon):  TGCTAGCAATTGATGGCACTTTCTTAACCGGTAAATACCAAGGTACATTGCTCATGGCAATAGGGGTCGACGCCGGGTTACATCTTGTTCCCTTGGCGTTTGCTCTGGTAGAGAAGGAGAATACATCCAACTGGGAGTGGTTCATCAACATGTTGAGAAACAAGTTGATCGGTCCTAATAGAGAAGTGTGTATAATTTCTGATAGACACCCTGGCATTTTGAACTCCATCATCCACATCATGCCTCATCACCTAACCATTCACCACAGGTGGTGCATGAGGCACTTCTGTGCTAACTTCTACACAGCTGGAGCTACGACTGATCAGATGAAAGATCTTGAGCGCATATGTCAAATAAATGAGAAAGCATTGTTCCTAGATGAGATAAAACGCCTCATGGGTGTGGTCGGAGAGAGGCCAAAGAAGTGGCTAGAAGACCATATGCCACTTAAGGTCAAATGGGCAAGAGCATTTGATACCAACGGGCGCTGAGTGTCCCACGGTCAGCGCCATTATGGCTGGCGCTGAGGTCCCAGCCCGCAAGCCATCCAGAAGGCTTGCTGGGCTGGGCCGTTTCGGCTGGGCTGGGCCATGGCCGAAACGGCCAAGTCCCACCTCAGCGCCAGGTGGGCTGGCGCTGAGTGTCCCACGGTCAGCGCCATTATGGCTGGCGCTGAGGTCCCAGCCCGCAAGCCATCCAGAAGGCTTGCTGGTCTGGGCCTTGGGGTTGCGGCCAAGTCCCACCTCAGCGCCAGTCCGGTTGGCGCTGAGGTAGCTGCCACGTCAGCTGGGGATCGGCCCGGCCGCCACGGTGGCACAATATCAGCGCCAGTCTCGTTGGCGCTGACACGGCCAACGTCAGCGCCAATGTGTTTGGCGCTGAGGCGACGGCCTATTTTTGGTTGAAGTTTTTGGCAGGGGTTAGTTTCGAAATAAGTTTTCTAAAAGGGTCAATTTGTCAAAAAAAAACGGCAAATTGAGTGCTTGCTGGGGACTTGAGAATGACTGGTGTTTATATATATGTATATATATTTAACCCAGCATGCTGCTATGGCCAACTGGAATCGGTATAGCTCATTACTCAAGACTTGTATTTGATTAATTTACTCTTCTTCCATACTGTACCTTGTGCTGCTTGCAGATTTATATTGAGGTTCAGTCAGTGGTCTTAAAGTGACGCCAAGTCATTTAAGGACTCGTATAATCTTTACTGTCTTATTGCCACGCTTTTAATTTCCCTCGTGTGTCATACTCAACATTATGTACATGGTTCATGCCATAACATATTTCATCTTTTTGTTTTGGCATGGTCCTTTAAGAGCACAAGGCTTCAGAAGCACAAGGAAGGGCAACACCGAAGGCAAGGAGAGCTTGATCACACCCCGCACCACCTATTCATGTCACAGGCAGCAGATTTCAGGTTGGCTAGCCTTTTTCCCTTTGTTTTTGCATGATGATATTTTAGCAAATCAAAGTGCCCGATTAATATTCCGATTCTACCACATCGCACACATCGGAAACAGAAAGAAAGAAGTTTCTCATTCCGTGCTTCATTTTTACTTCCTCCGTTTCGTTGTATTGTAGGGCGTTTTGATTTTTTTTTCCTGTAGTCAAACTTTTTTAAATTTAATCAAGTTTATAGAAAAATTTAGCAACATCTAAAACACGAAATTAGTTTTATTAAACTTAACATTGAATATATTTTGATAATATGTTTGTTTTGTATTGAAAATGCTACTATGTTTCTCTATAAACTCGGTCAAACTTATAAAAAATTAGTATGAAACAGATGGAGTATTATGTTTCCTTCTGTCTTGGATTTTCATGTTATGAATTGAAGTTGGTTGGTCAGTGTCAGGAAGAGAAACTTGAGGTTTTTGTTGAAATGTAAAGGGGTTTATTGATGTTTTCTTTTAGGTACCCCTTAATTAAATTAACTTTTACTCTTATTAGCTGACATGGTGTTATAAGTACAGGGTGGCACATGCTGAGGAACCAAAG | | | | | | |  |
| *OsFIONA1* | LOC_Os02g02880 | Amino acid sequence:  MGGGRKRRRRDGSEAPAIHPRNRYAAAAPDFASLASLYPSFAPFVSVSRGGRASIDFTDFAATRELTRVLLLHDHGVNWWIPDGQLCPTVPNRSNYIHWIEDLLSSDLIPPISSSNKTVRGFDIGTGANCIYPLLGASLLGWSFVGSDVTDVALEWAKKNVESNPQLAALVEIRNANKMSCSSESEAVDGEAARENTSKPVDGVLRSKPSILLGVVKDSESFDFCMCNPPFFESIEEAGLNPKTSCGGTAEEMVCPGGEQAFITRIIEDSVSLKNSFRWFTSMVGRKANLKILVSKVREAGVSVVKTTEFVQGQTARWGLAWSFIAPRKMVIRSSTPGKANYSFMLQGLRREYGAFQVLKSAESFFHASNLSCKTDSSLFSIDVTLSDEQAQAAMLHDESGSVEGNSTKLHSGVTGTSFRISVFEQMPGTLLVRGSLLNKALSGIFSSTFSQLEDTLKMEFLSKAR*  *Cis*-element sequence (2000 bp before start codon):  AGTGCCGTTTCGTCTTGTCTCCAATCTCCATGCACCTGGCTGTGGCGCCTAAAACCTGATCTCCAATTCATTTCAGGATCTAAACACAGAGGAGAAAATTATAAGAAGATGTGGAGAGTTTGTTGTGTTACATAAATACAAGATGACTTCATTTCCCTTTTAATAACAAAAAATAGTTGGTGAAGGAAATCTCACCATTGCAAAAAATGATCTTGAAACCCTCCAATTGCTCAACATGATAAATATTAAATTTCCCAAAACCGGTTTTATTAAGACATCCGTATTATATTTTGGGCAAAATGTTTTAATAAACATATTCCAATACAAAGTTCAAATACAACCTTGTCATCTTATATTCTATCTTATCCTTGCGCTGGATTATTGAAAAACTTAATACAATCCCCCGCTAATAATGATATAAAACAGTCCAACTGTTTAGGTCGGGCGTCGACAGCGGCTTATGTCGTTGTTCATCTCCGGCTTCGCCTTAGGATCTTACTCTTCCCACGGCCTTATGCGGTTTGTAGTTGTGTTTGCCTTAGGCTTTTGTGTCTTCTGTGTAAAATTTTTGCCGGTTTCACTTTAGCAAAACGCGCAGTTGTATGTTATTTGGCCCGGTTTTCCTTATAAACAGGGTCACACACTATCTTTCTAATATATTCGGCAATACTCTTGCCGCTTCCACTTAAAAAAGATATAAAACAGTCGATTACATATACACACGTACCTCCATACTGTTGATAATACTCGATGTTGATTGAAAATATGGTTGAAGTGTATATGTACCTAAAAAAAATATCTCATACTACCTTTTTTTTAACACAATTGCTGTTCATAAATTTGGTTAAATGAACAATGTAAGCCTACACAAAGTTTCACGAAACATTTAGGGCTGTTTGGTTGGTTGTCACACTTTGCCACGCCACACTTTAGCCATGCCACAGTTTTTTAGGCATGTGTTTGGTTCGCTGCCGCAATTGTGTTATACCACACTTTCTTAACTTTGGGACCCACATGTCATGCTCTCAAATTTAGAGCTAAGCATGACACACTTGTGGCCAACTATTTCTAAGCCACATTTATGCCACCATGCCACAACTAGCCTAAACTTAGTTATGGCAAGGTTAGGCATCAACCAAACATTTCCTTAGGCAGCATTCGAGAGGGTGAGAGAGAGTGAGTTTGTTTATTTCGTTTTCCGCGCGCACGTTTCCCAAACTATTAAACGGTGCGTTTTTTGCAAAAAAATTCTATAGGAAAGTTGCTTTAAAAAAATCATATTAATCTATTTTTGAAATTTAAAATAGTTAATACTCAATTAATCATGAGCTAATAGCTCACCTCGTTTTGCGTATCTTCCCAATCTCCTCAATCCCCTTCTCCTCAAACACACCCTAAGTGGTTTTACTCAAAATCTAAGGTTTCTTAAAGAAAACCACAATATTTGTGGTTTGCATGACAGATACCCTAAACTTGGTGGTTTATAAATTTTGCTTAATGATAAATGACACTGACATCTTTTTCATTTTTACCTGTATCACATTGTAGGATGAAAACACAAACCATGTCTTCATACTCTTGTTTTCTCAGTGAATTTCAAAAACACTATGCTATGGTCTATTTAGCAGGACTTCCAACTCCTGGAGTTGAGTTTAAAGCGGAGTTGTGGAGCTGAACACCGCTTCACCTTCTTAGTATAGTTTTTCATACCACTTAATCTACTCCAAACCATTTGGTTGAACTTCAGTTTCGAGATAGATGAAGCTGGAACTGAAGCCCTGCCAAACATATGTCTGCATTTGACAAAGATGAATTGTGGATGGGCCGTGATAGGGACTCGAGGCCCATGTAAAGATCTAAACGAAGGATTGGGCCGGAGAAAACCCCGAGCTTTTCTACCCGACCTGCGCCGCCGCGCCGCTCGCCGTCGCTTCCCCCGCGGCGCTTCGGGGCTCCGGCGATCTCTGCTCGAGCTCTGCCGCCGTTGGTCCATCCACGTC | | | | | | |  |
